# Triple Vectors Expand AAV Transfer Capacity in the Retina

**DOI:** 10.1016/j.ymthe.2017.11.019

**Published:** 2017-12-05

**Authors:** Andrea Maddalena, Patrizia Tornabene, Paola Tiberi, Renato Minopoli, Anna Manfredi, Margherita Mutarelli, Settimio Rossi, Francesca Simonelli, Jurgen K. Naggert, Davide Cacchiarelli, Alberto Auricchio

**Affiliations:** 1 Telethon Institute of Genetics and Medicine (TIGEM), Pozzuoli 80078, Italy; 2 Armenise/Harvard Laboratory of Integrative Genomics, TIGEM, Pozzuoli 80078, Italy; 3 Eye Clinic, Multidisciplinary Department of Medical, Surgical and Dental Sciences, Second University of Naples, Naples 80121, Italy; 4 The Jackson Laboratory, Bar Harbor, ME 04609, USA; 5 Medical Genetics, Department of Advanced Biomedicine, Federico II University, Naples 80131, Italy

**Keywords:** AAV, gene therapy, retina, large gene, triple AAV

## Abstract

Retinal gene transfer with adeno-associated viral (AAV) vectors holds great promise for the treatment of inherited retinal degenerations (IRDs). One limit of AAV is its transfer capacity of about 5 kb, which can be expanded to about 9 kb, using dual AAV vectors. This strategy would still not suffice for treatment of IRDs such as Usher syndrome type 1D or Alström syndrome type I (ALMS) due to mutations in *CDH23* or *ALMS1*, respectively. To overcome this limitation, we generated triple AAV vectors, with a maximal transfer capacity of about 14 kb. Transcriptomic analysis following triple AAV transduction showed the expected full-length products along a number of aberrant transcripts. However, only the full-length transcripts are efficiently translated *in vivo*. We additionally showed that approximately 4% of mouse photoreceptors are transduced by triple AAV vectors and showed correct localization of recombinant ALMS1. The low-photoreceptor transduction levels might justify the modest and transient improvement we observe in the retina of a mouse model of ALMS. However, the levels of transduction mediated by triple AAV vectors in pig retina reached 40% of those observed with single vectors, and this bodes well for further improving the efficiency of triple AAV vectors in the retina.

## Graphical Abstract

**Figure:**
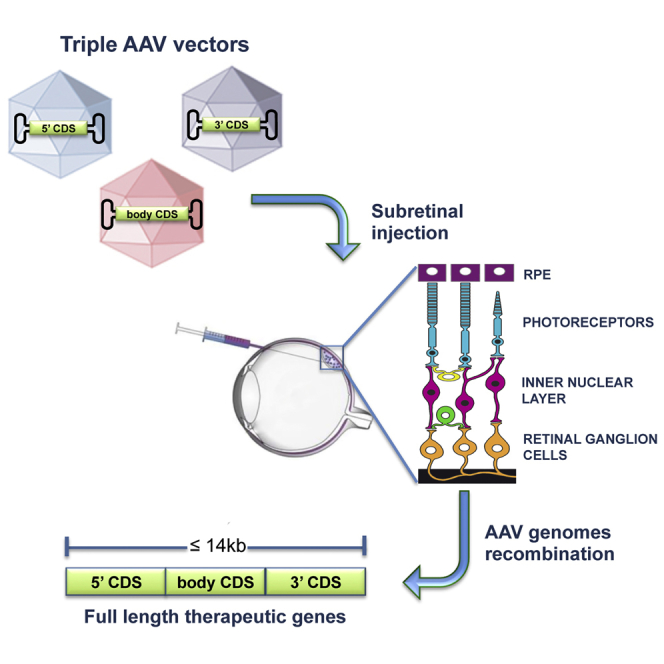

## Teaser

Maddalena et al. show that the limited DNA transfer capacity of adeno-associated viral (AAV) vectors can be expanded up to 14 kb with triple AAV vectors. The authors show triple AAV-mediated expression of both reporter and large therapeutic genes *in vitro* and in mouse and pig retina.

## Introduction

Inherited retinal degenerations (IRDs), with an overall global prevalence of 1/2,000,^1^ are a major cause of blindness worldwide. IRDs are mostly monogenic and are caused by mutations in genes mainly expressed in retinal photoreceptors (PRs; rods and cones) and to a lesser extent in retinal pigmented epithelium (RPE).^2^ To date, gene therapy based on adeno-associated viral (AAV) vectors represents the most promising therapeutic approach for IRDs. AAVs are (1) safe and effective when delivered subretinally in patients with Leber congenital amaurosis type 2, a severe form of inherited childhood blindness^3^, ^4^, ^5^, ^6^, ^7^, ^8^ and (2) to date, the most effective gene transfer vector for PRs in addition to RPE.^9^, ^10^, ^11^, ^12^, ^13^, ^14^, ^15^ However, the relatively small DNA packaging capacity of AAV, which is considered to be restricted to the size of the parental genome (4.7 kb),^16^, ^17^, ^18^, ^19^ prevents its application from the treatment of those IRDs caused by mutations in genes having a coding sequence (CDS) larger than 5 kb, such as Stargardt disease (STGD1; MIM_248200), Usher syndrome types IB (USH1B; MIM_276900) and 1D (USH1D; MIM_601067), or Alström syndrome (ALMS; MIM_203800), among others.

Dual AAV vectors^20^, ^21^, ^22^, ^23^ have been developed to overcome the limited-cargo capacity of AAV by splitting a large transgene expression cassette in two separate halves, each independently packaged in a single normal-size (<5 kb) AAV vector.^20^, ^21^, ^23^, ^24^ The reconstitution of the full-length expression cassette is achieved upon co-infection of the same cell by both dual AAV vectors followed by inverted terminal repeat (ITR)-mediated tail-to-head concatemerization of the 5′ and 3′ genomes and/or followed by splicing (dual AAV *trans*-splicing vectors [TS]);^20^, ^21^, ^22^, ^25^ homologous recombination between overlapping regions contained in both the 5′ and 3′ genomes (dual AAV overlapping vectors)^21^ or a combination of the two (dual AAV hybrid vectors), where the directional concatemerization can be favored by the presence of highly recombinogenic sequences.^23^ We have recently shown that both dual AAV TS and hybrid AK vectors (that contain the short AK recombinogenic region from the F1 phage) efficiently transduce mouse PRs and RPE, and rescue mouse models of STGD1 and USH1B^26^ caused by mutations in *ABCA4* (ATP-binding cassette subfamily A member 4; CDS, 6.8 kb) and *MYO7A* (MYOSIN VII A; CDS, 6.6 kb), respectively. Robust *MYO7A* reconstitution by dual AAV vectors was confirmed in the mouse retina by Dyka et al.,^27^ while several examples of dual AAV systems for minidystrophin (CDS, 6.2 kb) expression in mouse muscle are available.^23^, ^28^ The efficacy of dual AAV vectors, with a maximum transfer capacity of around 9 kb, opens the venue for further expansion to triple AAV vectors with a theoretical transfer capacity of around 14 kb.

Triple AAV vectors, in principle, would enable the development of gene therapies for IRDs due to mutations in genes whose CDS is larger than 9 kb (herein referred to as large genes). Among those IRDs are (1) USH1D, a severe form of autosomal recessive blindness-deafness which accounts for 19%–35% of cases of Usher syndrome type 1^29^, ^30^, ^31^ and which is caused by mutations in *CDH23* (CDS, 10.1 kb), which encodes for the Cadherin-related family member 23^32^, ^33^ and (2) ALMS, an autosomal recessive condition, with a prevalence of less than one per million^34^, and characterized by a combination of features including obesity, insulin resistance, and retinal dystrophy. ALMS is caused by mutations in *ALMS1* (CDS, 12.5 kb), which encodes for a ciliary/centrosomal protein thought to play a key role in transport along the PR axoneme.^34^, ^35^, ^36^, ^37^, ^38^ Triple AAV vectors have previously been exploited to reconstruct full-length *dystrophin* (CDS, 11.1 kb) in the muscle of dystrophic mice.^39^, ^40^ Low levels of full-length dystrophin expression were obtained by both TS^40^ and hybrid systems.^39^ These results demonstrated great potential for testing triple AAV vectors in the retina to restore *CDH23* and *ALMS1* gene expression. Indeed, the enclosed and small subretinal space should favor co-infection and transduction of the same cell by three independent AAV vectors.

## Results

### Generation of Single and Triple AAV Vectors

**Figure 1:**
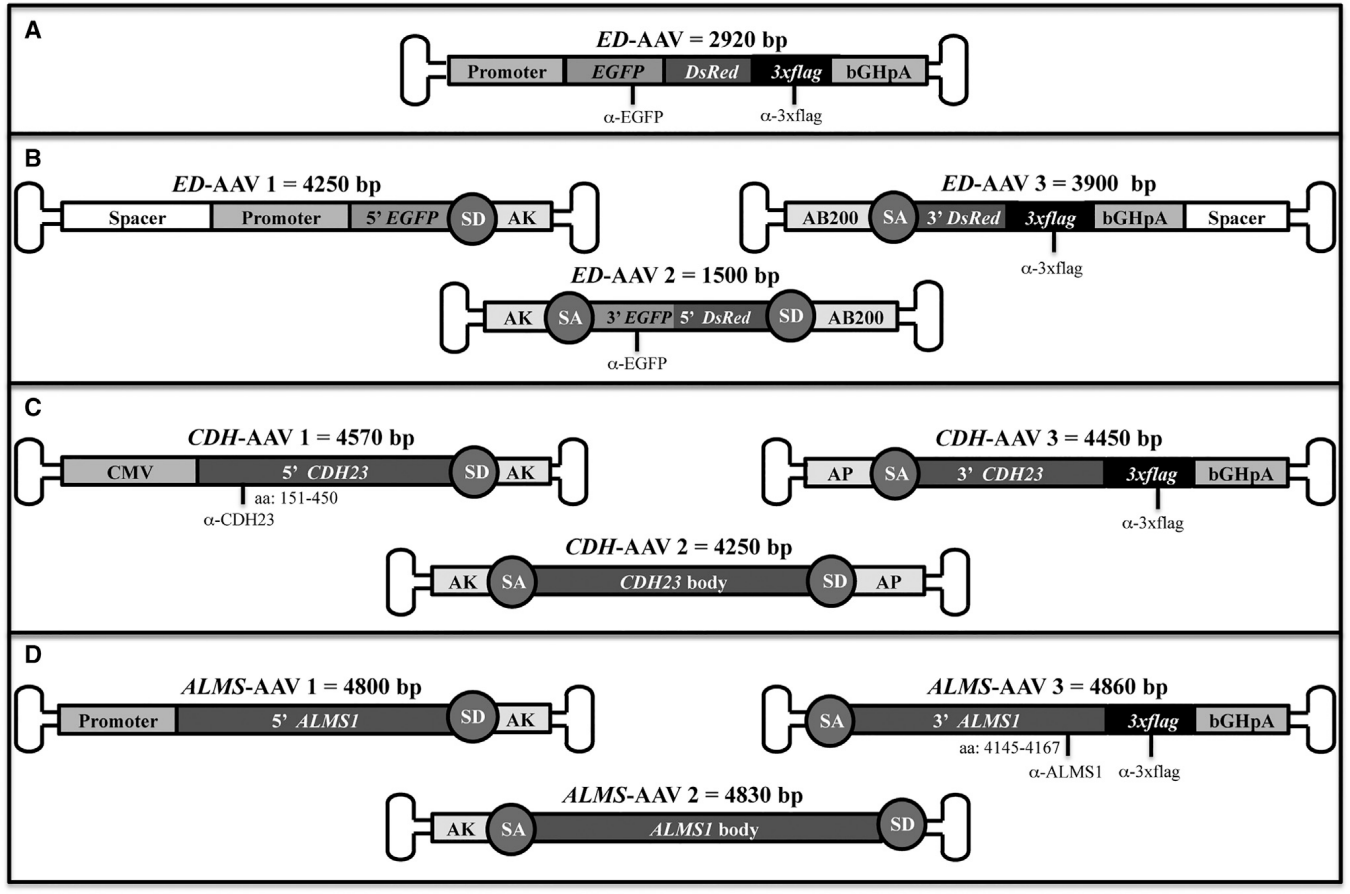
Schematic Representation of Single and Triple AAV Vectors Single *ED*-AAV (A); triple *ED*-AAVs (B); triple *CDH-*AAVs (C); triple *ALMS-*AAVs (D). The position of the epitopes recognized by the antibodies used in this study is indicated. The vector genome size is depicted above each vector. bGHpA, bovine growth hormone polyadenylation signal; SD, splicing donor signal; SA, splicing acceptor signal; AP, alkaline phosphatase recombinogenic region; AK, F1 phage recombinogenic region; AB200, *ABCA4* recombinogenic region; *3xflag*, triple flag tag; CMV, cytomegalovirus; α-EGFP, anti-EGFP antibodies; α-3xflag, anti-3xflag antibodies; α-CDH23, anti-CDH23 antibodies; α-ALMS1, anti-ALMS1 antibodies.

In order to test transduction efficiency mediated by triple AAV vectors, we generated a reporter protein by fusing the CDS of *EGFP* to that of *Discosoma red fluorescent protein* (*DsRed*) (herein referred to as *ED*) separated by a 13 amino acid spacer. A triple flag tag (*3xflag*) was added at the 3′ end of *ED* CDS (Figure 1A), which was placed under the control of (1) the ubiquitous cytomegalovirus (CMV) promoter, (2) the PR-specific human interphotoreceptor retinoid-binding protein (IRBP) promoter,^41^, ^42^ or (3) the RPE-specific vitelliform macular dystrophy 2 (VMD2) promoter.^43^ The *ED* cassettes were either packed in a single AAV vector or split in three parts, each packed in a different AAV vector (Figure 1B and Materials and Methods) from here called *ED*-AAV 1, *ED*-AAV 2, *ED*-AAV 3. Upon HEK293 transfection with plasmids expressing ED, the ED spontaneous fluorescence appeared weaker than the fluorescence detected when single EGFP- or single DsRed-expressing plasmids were used (Figure S1).

Similarly, *CDH23*-*3xflag* and *ALMS1*-*3xflag* were split in three parts each cloned in a separate AAV vector (Figures 1C and 1D and Materials and Methods) from here called *CDH*-AAV 1, *CDH*-AAV 2, *CDH*-AAV 3, and *ALMS*-AAV 1, *ALMS*-AAV 2, *ALMS*-AAV 3. *CDH*-AAV 1 and *ALMS*-AAV 1 included the ubiquitous CMV and either the short CMV (shCMV) or the PR-specific human G protein-coupled receptor kinase 1 (GRK1) promoters, respectively. For the *in vitro* experiments, we generated AAV2/2 vectors, which efficiently transduce HEK293 cells.^44^ In the experiments performed in the mouse and pig retinas, we used AAV2/8 vectors, which efficiently transduce RPE and PRs^9^, ^10^, ^11^ but poorly infect HEK293 cells.

### Triple *ED*-AAV Vectors Efficiently Transduce HEK293 Cells

**Figure 2:**
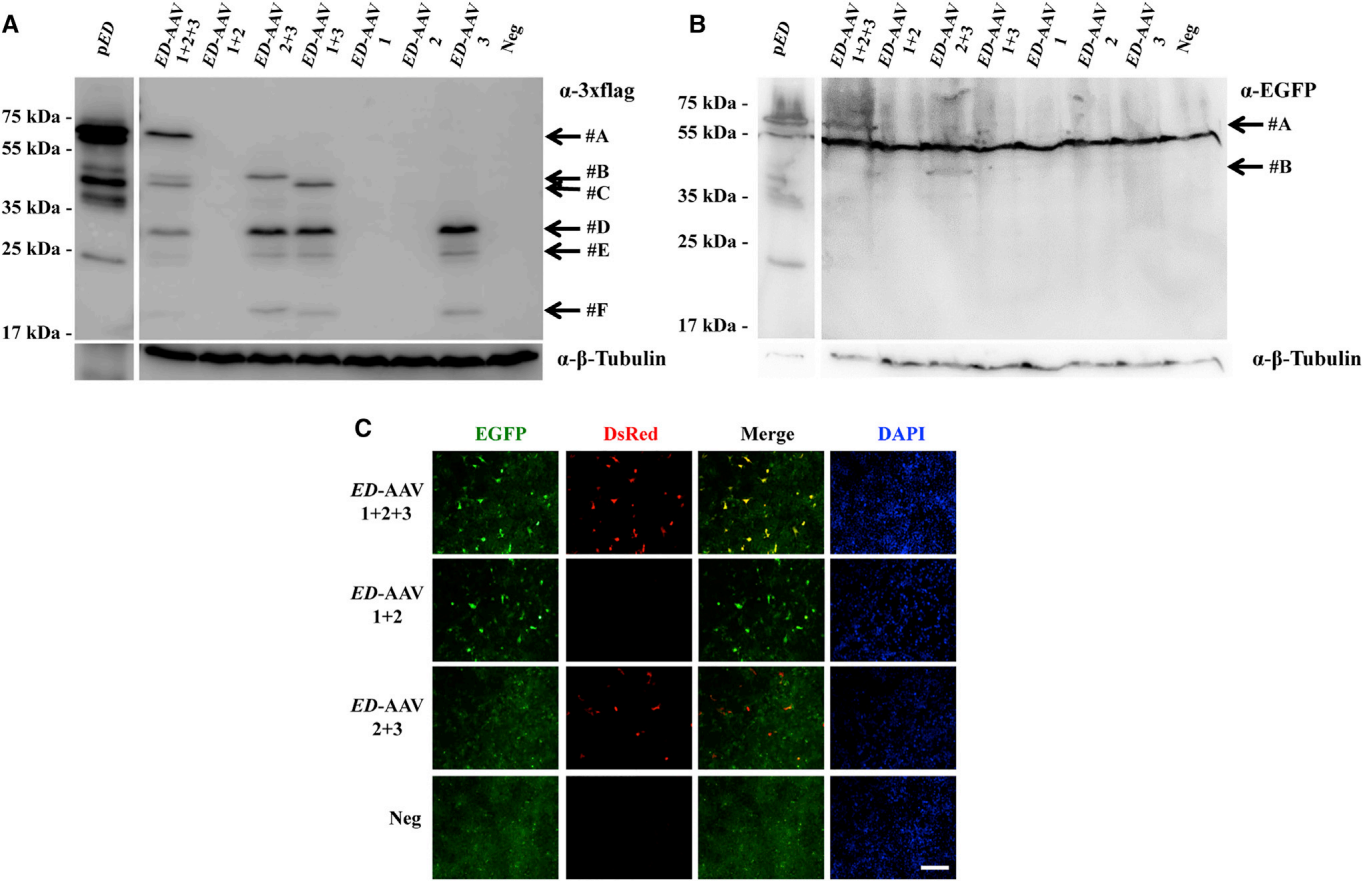
Triple *ED*-AAV Vectors Efficiently Transduce ED *In Vitro* (A and B) Western blot (WB) analysis of lysates from HEK293 cells either transfected with p*ED* or infected with triple AAV2/2 vectors encoding for ED and stained with α-3xflag (A) and α-EGFP (B) antibodies. The arrows on the right indicate the following protein products: #A, ED full-length protein; #B, from AAV 2 + 3; #C, from AAV 1 + 3; #F, from AAV 3; #D and #E, aggregates of AAV 3. α-3xflag, WB with anti-3xflag antibodies; α-EGFP, WB with anti-EGFP antibodies; α-β-Tubulin, WB with anti-β-Tubulin antibodies, used as loading control. Neg, not infected cells. The molecular weight ladder is depicted on the left; 20 and 200 μg of proteins for transfected and infected samples, respectively, were loaded. The WB images are representative of n = 4 independent experiments. (C) Fluorescent analysis of HEK293 cells infected with triple AAV2/2 vectors encoding for ED. The scale bar (200 μm) is depicted in the figure. DsRed, Discosoma red fluorescent protein; Merge: overlay of EGFP and DsRed images.

First, we evaluated the efficiency of triple *ED*-AAV-mediated large gene transduction *in vitro* by infecting HEK293 cells with AAV2/2 vectors at an MOI of 5 × 10^4^ genome copies (GC)/cell of each vector. Seventy-two hours after infection, cell lysates were analyzed and the ED expression was evaluated by western blot (WB) using anti-3xflag (Figure 2A) or anti-EGFP (Figure 2B) antibodies (please see their specific epitope localization in Figure 1), by direct microscope imaging (Figure 2C) and by cytofluorimetry. As shown in Figures 2A and 2B, full-length proteins (#A) of the expected size (≈60 kDa) were detected only following co-infection with the three *ED*-AAV vectors. Various shorter products were detected by the anti-3xflag antibodies when *ED*-AAV 3, which includes the 3xflag tag, was added to the infection mix (Figure 2A). These include (1) a product that derives from *ED*-AAV 2 + 3 (#B); (2) a product that might derive from the concatemerization of *ED*-AAV 1 + 3 and the use of an alternative ATG in frame with the 3xflag (#C); (3) a product from *ED*-AAV 3 alone (#F); and (4) products that might represent aggregates of #F (#D and #E). WB with anti-EGFP antibodies (Figure 2B) confirmed the presence of both full-length ED (#A) when the 3 *ED*-AAV vectors were used and the shorter #B product in the *ED*-AAV 2 + 3 sample. Interestingly, the relative amount of the truncated products decreases when the 3 *ED*-AAV vectors are used.

ED expression was also evaluated by direct imaging of native fluorescence (Figure 2C). Double EGFP and DsRed-positive cells could be detected only when the 3 *ED*-AAVs were used. Cells that are positive for either EGFP or DsRed alone were detected when *ED*-AAV 1 + 2 or *ED*-AAV 2 + 3 were respectively used, but they were almost absent in the *ED*-AAV 1 + 2 + 3 samples.

The efficiency of triple *ED*-AAVs transduction was then quantified by cytofluorimetry, which showed that double EGFP and DsRed-positive cells were present above background only in single *ED*-AAV (46.69% ± 5.66%; n = 4) and in *ED*-AAV 1 + 2 + 3 (2.78% ± 0.84%; n = 3) samples. Therefore, the efficiency of triple *ED*-AAVs transduction is 5.96% ± 1.95% of that of the corresponding single vector.

### Triple *CDH-* and *ALMS*-AAV Vectors Efficiently Transduce HEK293 Cells

HEK293 cells were infected with the various combinations of *CDH-* and *ALMS*-AAV2/2 vectors at an MOI of 5 × 10^4^ GC/cell and analyzed 72 hr post-infection. The expression of full-length and truncated products of CDH23 and ALMS1 was evaluated at both transcriptional and translational levels.

**Figure 3:**
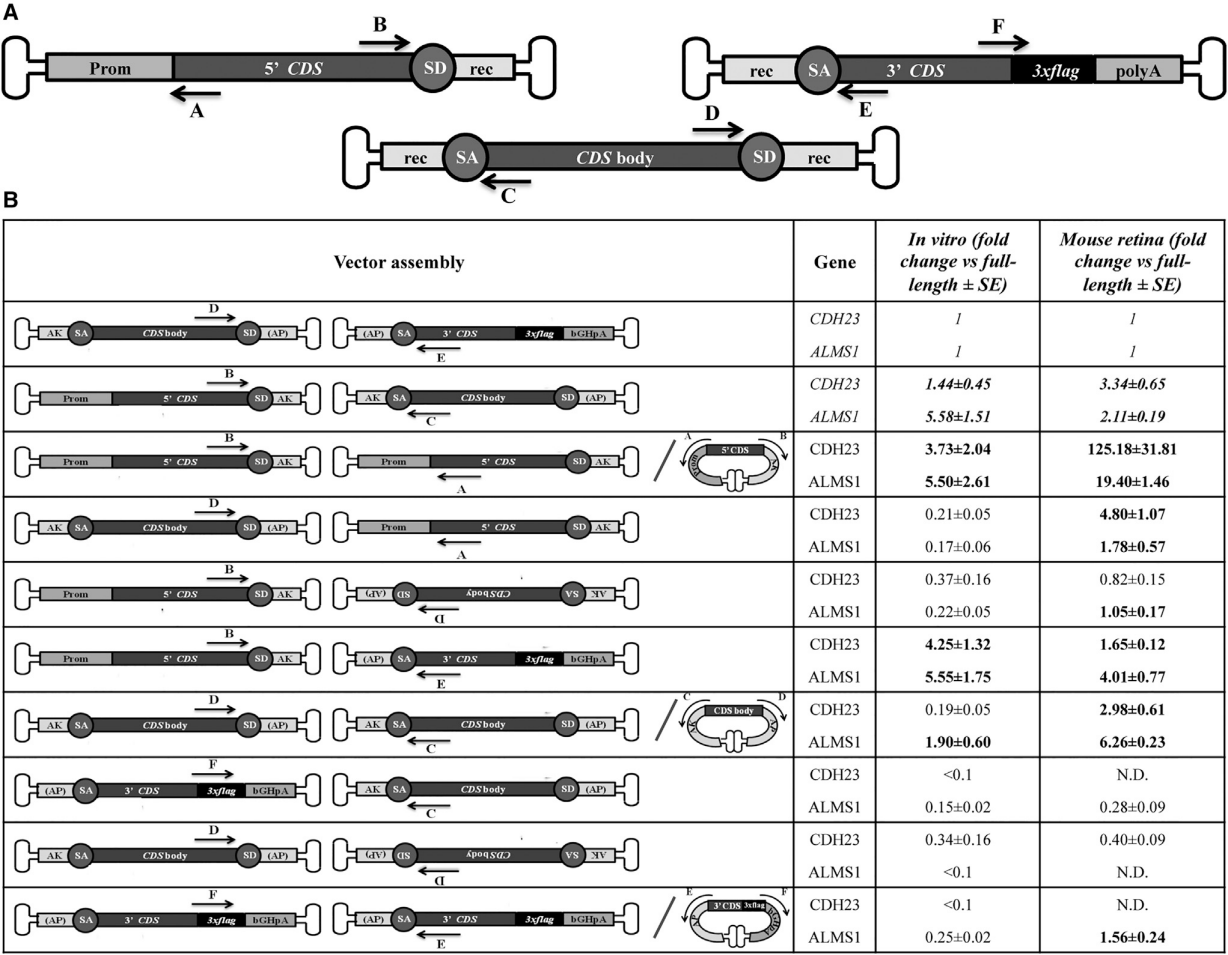
Transcriptomic Analysis following Transduction with Triple AAV Vectors (A) Schematic representation of triple *CDH* and *ALMS*-AAV vectors with primers used for real-time qPCR (A→F). Prom, promoter; CDS, coding sequence; polyA, polyadenylation signal; *3xflag*, triple flag tag; SD, splicing donor signal; SA, splicing acceptor signal; rec, recombinogenic region. (B) Results of the real-time qPCR analysis performed on cDNAs from HEK293 cells or mouse eyecups treated with either *CDH*- or *ALMS*-AAV 1 + 2 + 3 vectors. The left column shows the vector assembly predicted on the basis of the set of primers used. The two columns on the right show the abundance of the PCR products relative to full-length detected by the D + E primers. Only products with a relative abundance >0.1 are shown. Highlighted in italic is the relative abundance of PCR products resulting from the desired AAV directional concatemerization. Highlighted in bold are the PCR products with a relative abundance >1.

cDNAs from HEK293 infected cells were analyzed by real-time qPCR. For each transgene, six primers (A→F) were used, each annealing to one extremity of the three CDSs separately included in each of the triple vectors (Figure 3A). First, we observed that the real-time qPCR amplification using primers D (located at the 3′ end of vector 2) and E (located at the 5′ of vector 3) following infection with AAV 2 + 3 is significantly lower than the one obtained when AAV 1 + 2 + 3 are used (2% for *CDH23* and 20% for *ALMS1*). This means that amplification with this set of primers can be used as a close estimate of full-length transcript following concatemerization of the three vectors. For simplicity, from now on, such measurement will be set to 1 as reference, and the abundance of the other products will be presented as fold change relative to the reference. To detect the transcripts from all possible triple AAV concatemers, the A→F primers were tested in all possible combinations. The sets of primers that generated a PCR product with a relative abundance >0.1 compared to full-length are reported in Figure 3B, along with a schematic representation of the corresponding predicted AAV concatemer. For both *CDH23* and *ALMS1* transgenes, amplification of products with a relative abundance higher than full-length was obtained with the following primer couples: B + C, A + B, B + E, and, only in the case of *ALMS1*, C + D (shown in bold in Figure 3B, “*in vitro*” column). Direct sequencing of real-time qPCR products revealed that, upon correct assembly of the triple *CDH*- or *ALMS*-AAV, correct splicing occurs (data not shown).

As the real-time qPCR analysis cannot reveal the generation of alternative splicing within the three CDSs, we additionally performed a targeted RNA-seq analysis with *de novo* annotation of the two transgenes. The analysis annotated the presence of the full-length transcript in both CDH23 (Figure S2A) and ALMS1 (Figure S2B) samples. Similar to what we observed in the real-time qPCR analysis, the most represented products derive from the concatemerization of AAV 1 + 3. It is interesting to note that the CDS of *CDH*-AAV 3 seems to generate several isoforms as a consequence of alternative splicing.

**Figure 4:**
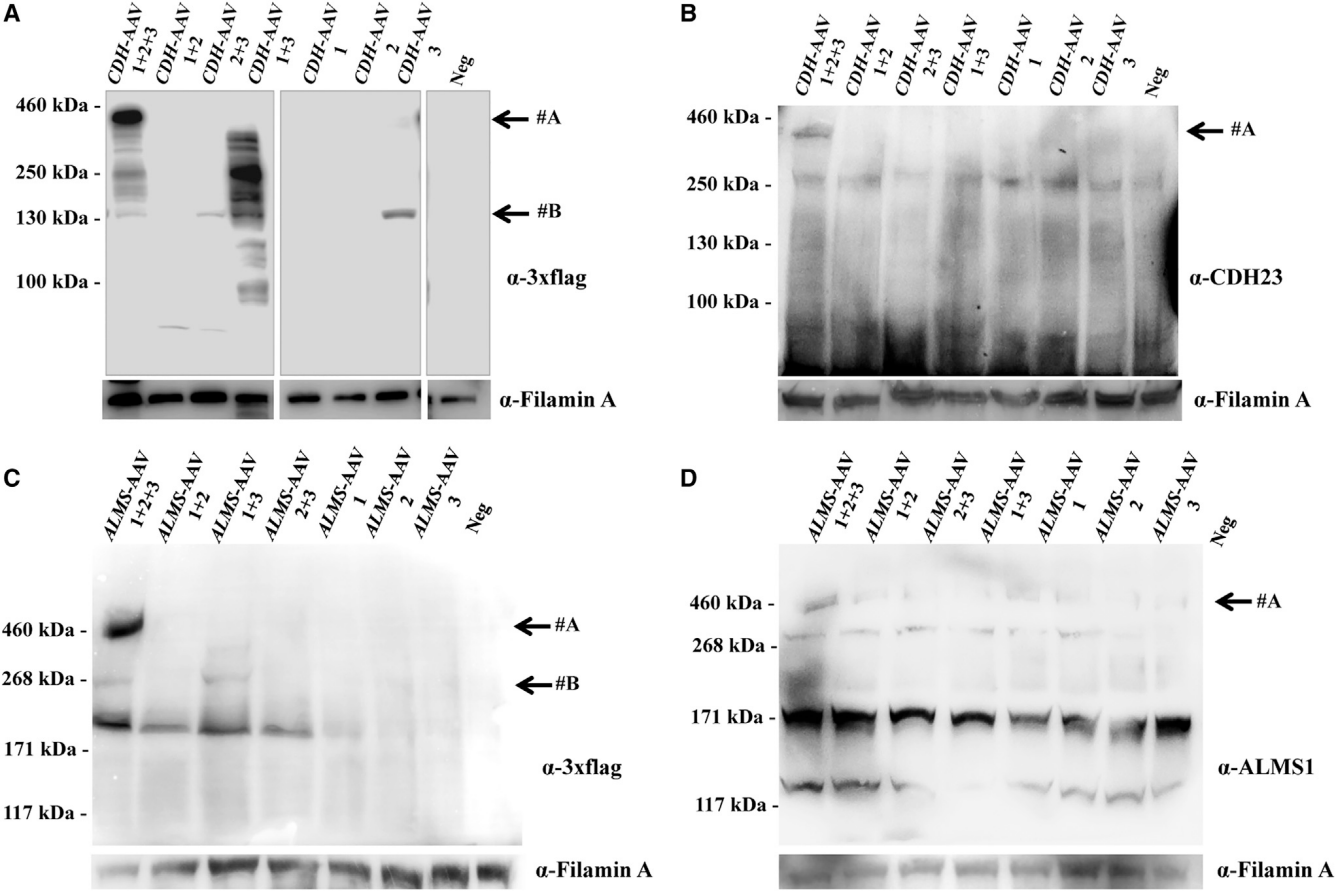
Triple *CDH-* and *ALMS*-AAV Vectors Efficiently Transduce CDH23 and ALMS1 *In Vitro* (A and B) WB analysis of lysates from HEK293 cells infected with triple AAV2/2 vectors encoding for CDH23 and incubated with either anti-3xflag (A) or anti-CDH23 (B) antibodies. The arrows on the right indicate the following protein products: #A, full-length CDH23 protein; #B, from AAV 3. (C and D) WB analysis of lysates from HEK293 cells infected with triple AAV2/2 vectors encoding for ALMS1 and incubated with either anti-3xflag (C) or anti-ALMS1 (D) antibodies. The arrows on the right indicate the following protein products: #A, full-length ALMS1 protein; #B, from AAV 1 + 3. α-3xflag, WB with anti-3xflag antibodies; α-CDH23, WB with anti-CDH23 antibodies; α-ALMS1, WB with anti-ALMS1 antibodies; α-Filamin A, WB with anti-Filamin A antibodies, used as loading control. Neg, cells infected with control AAV2/2-CMV-*EGFP* vectors. The molecular weight ladder is depicted on the left, 100 (A), 250 (B), or 200 μg (C and D) of proteins were loaded.

Following detailed transcriptional characterization of HEK293 cells infected with triple AAV vectors, we tested the presence of full-length and truncated CDH23 and ALMS1 protein products. WB analysis of lysates from infected cells was performed using antibodies (please see their specific epitope localization in Figure 1) directed to either the 3xflag tag (Figures 4A and 4C) or CDH23 and ALMS1 (Figures 4B and 4D). In all cases, full-length protein (#A) was detected with both anti-3xflag and anti-CDH23 or anti-ALMS1 antibodies only when the three *CDH*- (Figures 4A and 4B; ≈360 kDa) or *ALMS*-AAVs (Figures 4C and 4D; ≈461 kDa) were used. Please note that the shape of the ALMS1 bands in the lanes *ALMS*-AAV 1 + 2 + 3 may be due to the challenging migration of such high molecular weight proteins in the SDS-PAGE. In addition to the full-length proteins, several shorter products were observed: (1) a truncated product (#B) which corresponds to CDH23 C terminus was detected with anti-3xflag antibodies when *CDH*-AAV 3 was included in the infection mix (Figure 4A); (2) only in the sample infected with *CDH*-AAV 1 + 3, a smear of bands was observed that was greatly reduced when the three *CDH*-AAV vectors were included in the infection mix (Figure 4A); (3) a truncated product of ≈270 kDa (#B) was detected with anti-3xflag antibodies when *ALMS*-AAV 1 + 3 was included in the infection mix (Figure 4C). The two bands of ≈171 and ≈117 kDa present in all lanes of the WB shown in Figure 4D are presumably due to aspecific antibody binding.

### Subretinal Administration of Triple *ED*-AAVs Results in Full-Length Transgene Expression in Mouse and Pig Retina

**Figure 5:**
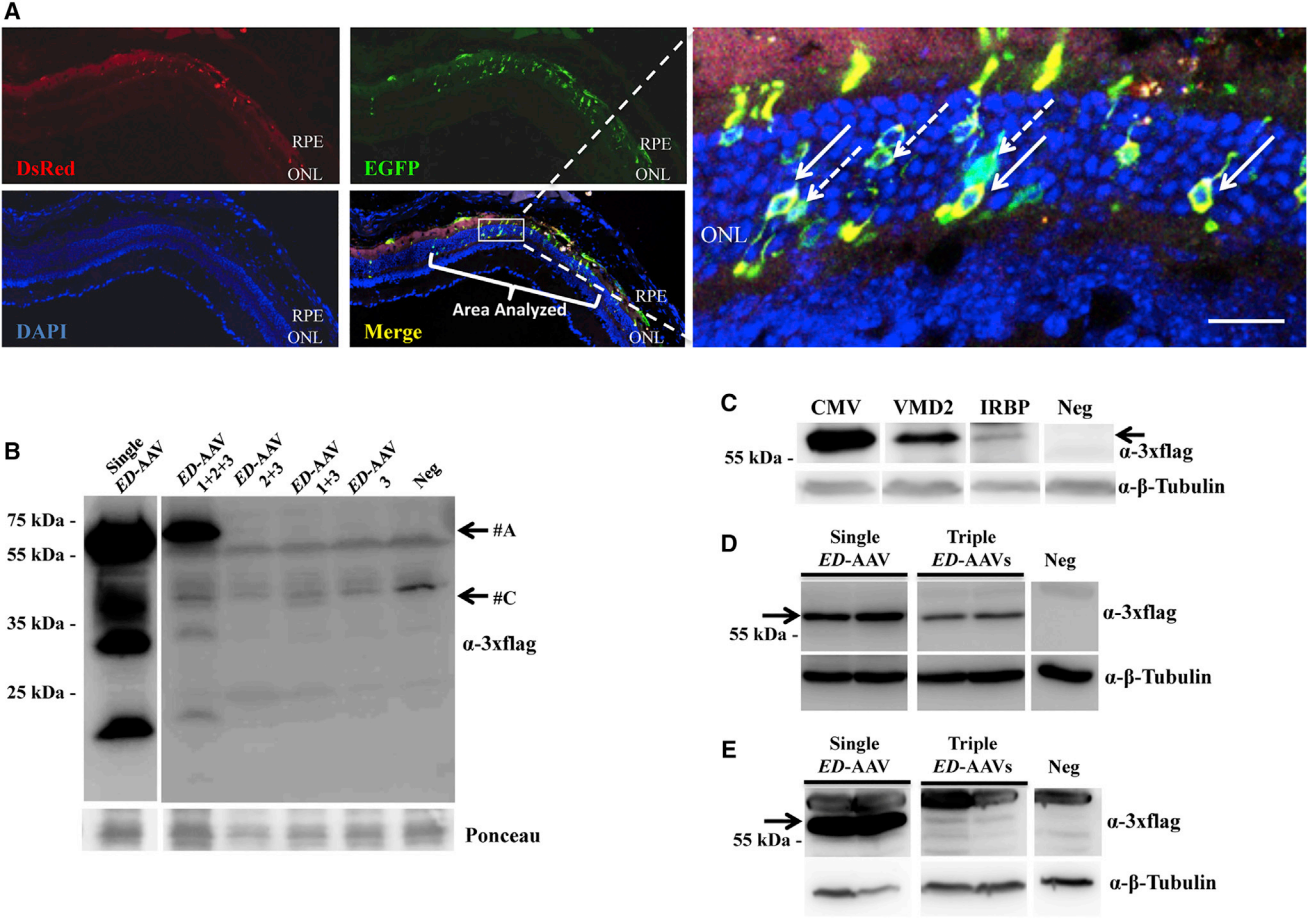
Triple AAV Vectors Drive Full-Length Protein Expression in the Mouse Retina (A) Fluorescence analysis of retinal cryosections from C57BL/6J mice 2 months following subretinal injection of triple *ED*-AAV2/8 vectors under the control of the ubiquitous CMV promoter. The pictures with insets at higher magnification are representative of n = 5 injected eyes. Full arrows indicate EGFP and DsRed double-positive PRs. Dashed arrows indicate EGFP-only positive PRs. The scale bar (20 μm) is depicted in the figure. DsRed, Discosoma red fluorescent protein; Merge, overlay of EGFP, DsRed, and DAPI images; RPE, retinal pigmented epithelium; ONL, outer nuclear layer. (B) Western blot (WB) analysis of truncated products in eyecup lysates from C57BL/6J mice 2 months following subretinal injection of the combinations of *ED*-AAVs. The arrows indicate the following products: #A, ED full-length protein; #C, from AAV 1 + 3. (C) WB analysis of lysates from C57BL/6J eyecup 2 months following subretinal injection of triple AAV2/8 vectors encoding for ED under the control of the ubiquitous CMV, the RPE-specific VMD2, and the PR-specific IRBP promoters. The arrow indicates the full-length protein. (D) WB analysis of eyecup lysates from C57BL/6J mice 2 months following subretinal injection of single and triple AAV2/8 vectors encoding for ED under the control of the ubiquitous promoter CMV. (E) WB analysis of eyecup lysates from C57BL/6J mice 2 months following subretinal injection of single and triple AAV2/8 vectors encoding for ED under the control of the PR-specific promoter IRBP. CMV, cytomegalovirus; VMD2, vitelliform macular dystrophy 2; IRBP, human interphotoreceptors retinoid binding proteins. α-3xflag, WB with anti-3xflag antibodies; Ponceau, staining with Ponceau, used as loading control; α-β-Tubulin, WB with anti-β-Tubulin antibodies, used as loading control. Neg, lysates of eyecups following injection with PBS. The molecular weight ladder is depicted on the left; 100 (D), 150 (B and C), and 200 (E) μg of proteins were loaded.

We hypothesized that the enclosed and small subretinal space should favor co-infection and transduction of the same cell by three independent AAV vectors. To evaluate this, we injected subretinally triple CMV-*ED*-AAV2/8 vectors (dose of each vector/eye, 4.2 × 10^9^ GC) in 4-week-old C57BL/6J mice (n = 7 eyes). Eyes were harvested 2 months later, and we analyzed, on retinal cryosections, the number of PR cells expressing ED (Figure 5A). We found, in the transduced area of 5 out of 7 eyes, that 2.9% ± 0.5% were positive for EGFP alone (which derives from concatemerization of only *ED*-AAV 1 + 2) and 3.6% ± 0.3% were positive for both EGFP and DsRed (which derives from concatemerization of *ED*-AAV 1 + 2 + 3). As no PRs were positive for DsRed alone (which derives from *ED*-AAV 2 + 3), we conclude that the co-expression of EGFP-DsRed indicates full-length protein expression deriving from proper directional concatemerization of *ED*-AAV 1 + 2 + 3.

To confirm by WB analysis the retinal expression of full-length ED protein as well as to detect potential byproducts from *ED*-AAV 1 + 3 that could not be viewed on retinal histological sections, the following combinations of triple *ED*-AAVs (dose of each vector/eye, 4.2 × 10^9^ GC) were injected subretinally in 4-week-old C57BL/6J mice: *ED*-AAV 1 + 2 + 3 (n = 8 eyes); *ED*-AAV 2 + 3 (n = 8 eyes); *ED-*AAV 1 + 3 (n = 6 eyes); *ED*-AAV 3 (n = 6 eyes); single *ED*-AAV (n = 6 eyes). Eyecups were harvested 2 months later, and WB analysis was performed using anti-3xflag antibodies. As shown in Figure 5B, apart from the full-length protein (#A), when the 3 *ED*-AAV vectors were included in the mix, an additional faint band of lower molecular weight was detected (#C), which has a molecular weight similar to that detected *in vitro* in the *ED*-AAV 1 + 3 sample (#C) (Figure 2A). However, this product was present only in one out of six eyes injected with *ED*-AAV 1 + 3, confirming that this is a rare event which was not detected in retinal histological sections (Figure 5A). The other bands present in *ED*-AAV 1 + 2 + 3 sample, which are also present in the samples injected with single *ED*-AAV, might represent degradation products of the full-length ED protein.

We then investigated whether triple AAV transduction occurs in different retinal cell types. Toward this end, 4-week-old C57BL/6J mice were injected subretinally with three sets of triple *ED*-AAV vectors, each with a different promoter: CMV, IRBP, and VMD2 at the doses of 4.2 × 10^9^, 3.7 × 10^9^, and 2.4 × 10^9^ GC/each vector/eye, respectively. Animals were sacrificed 2 months post-injection, and protein lysates from eyecups were analyzed by WB, which showed that full-length ED reconstitution and expression can be driven by all three promoters, although with different efficiencies. Subretinal injection of triple VMD2-*ED*-AAV vectors, which includes the RPE-specific VMD2 promoter, displayed strong protein expression in 10/10 analyzed eyes. The ubiquitous CMV promoter resulted in slightly higher levels of ED expression compared to VMD2 in 13/18 (72%) analyzed eyes. The IRBP promoter, specific for PR expression, resulted in faint ED expression in 14/19 (74%) analyzed eyes (Figure 5C).

To assess if the weak PR transduction observed with the IRBP promoter was due to poor transcriptional activity of the promoter in the PRs or limited recombination upon co-infection of the three vectors, we compared the expression of the ED protein encoded by the triple AAV vectors to the expression by a single AAV vector containing the same expression cassette (Figures 5D and 5E; Figures S3 and S4). The experiment was performed with both the CMV (Figures 5D and S3) and IRBP (Figures 5E and S4) promoters, and ED protein expression was evaluated by WB analysis with anti-3xflag antibodies. WB band intensity showed that, in the case of the CMV promoter, the level of ED expression obtained with the triple AAV vectors was 27% ± 6% of the one obtained with the single AAV vector (n of eyes injected with single AAV = 12; n of eyes injected with triple AAVs = 14), while this decreased to 2% ± 1% when IRBP promoter was used (n of eyes injected with single AAV = 6; n of eyes injected with triple AAVs = 8), thus suggesting that multiple AAV transduction, rather than the promoter strength, impacts on mouse PR transduction, as also previously observed with dual AAV vectors.^45^

**Figure 6:**
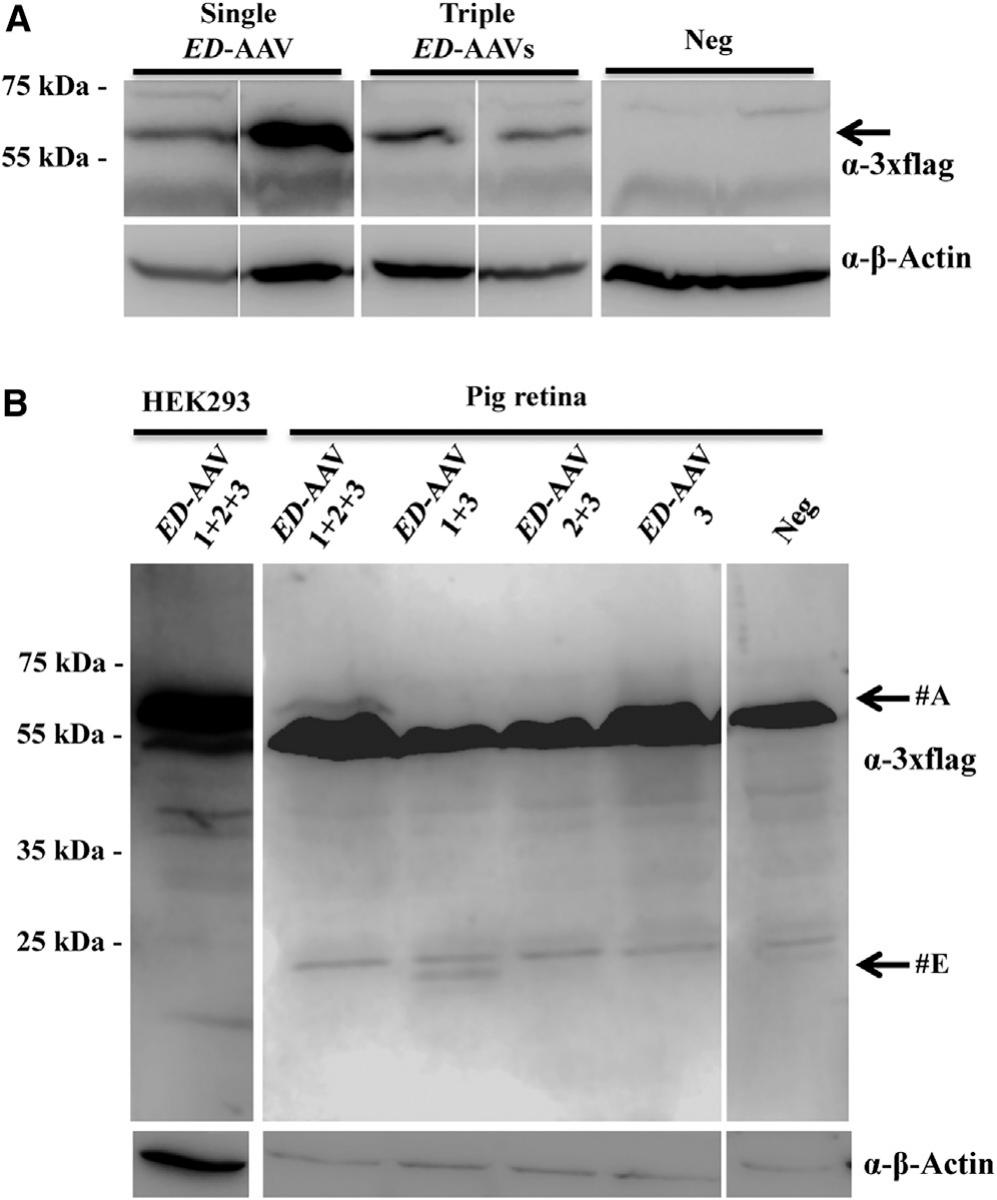
Triple AAV Vectors Drive Full-Length Protein Expression in the Pig Retina (A) Western blot (WB) analysis of lysates from Large White pig retina 2 months following subretinal injection of either single or triple AAV2/8 vectors encoding for ED under the control of the PR-specific promoter IRBP. The arrow indicates the full-length protein. (B) WB analysis of truncated products in lysates from Large White pig retina 2 months following subretinal injection of the combinations of triple *ED*-AAVs. Lysates of HEK293 cells infected with an *ED*-AAV 1 + 2 + 3 are shown on the left as positive control. The arrows indicate the following products: #A, ED full-length protein; #E, probably from AAV 3. α-3xflag, WB with anti-3xflag antibodies; α-β-Actin, WB with anti-β-Actin antibodies, used as loading control. Neg, lysates of retinas following injection with PBS. The molecular weight ladder is depicted on the left; 200 μg of proteins were loaded.

We then evaluated the efficacy of the triple *ED*-AAV vectors at transducing PRs in the pig retina. The pig retina is an excellent model to evaluate viral vector transduction characteristics because of its size, which is similar to the human retina, and because it is enriched with cones that are concentrated in a streak-like region similar to what happens in the primate macula.^10^ We injected subretinally Large White pigs with single and triple AAV2/8 vectors encoding ED protein under the transcriptional control of the PR-specific promoter IRBP (dose of each vector/eye, 1 × 10^11^ GC), and found two out of three positive eyes in both groups. WB band intensity quantification showed that ED protein expression with triple AAV vectors was 39% ± 17% (n = 2) of that observed with a single AAV vector (Figure 6A). Moreover, no truncated products were detected by WB analysis with anti 3xflag antibodies of neural retina lysates following subretinal injection of *ED*-AAV 1 + 2 + 3 (Figure 6B). A shorter product was observed in the *ED*-AAV 1 + 3 sample (#E), which is similar to the one observed in infected cells (#E) (Figure 2A). Overall, these data suggest that triple AAV vectors transduce pig PRs more efficiently than mouse, as previously observed with dual AAV vectors.^26^

### Expression of Therapeutic Transgenes Mediated by Triple AAV Vectors in the Mouse Retina

We then tested whether subretinal administration of triple AAV2/8 vectors results in expression of *CDH23* and *ALMS1*, which are both mutated in syndromic forms of retinitis pigmentosa.^32^, ^33^, ^34^, ^35^, ^36^, ^37^

We injected subretinally 4-week-old C57BL/6J mice with either triple CMV-*CDH*-AAV (dose of each vector/eye, 2 × 10^9^ GC) or triple shCMV-*ALMS*-AAV vectors (dose of each vector/eye, 2 × 10^9^ GC). Animals were sacrificed 2 to 3 months post-injection, and transgene expression was evaluated by both real-time qPCR and WB. Under the assumption that the PCR product amplified with the D + E primer couple derives mostly from full-length transcripts, we compared the levels of triple AAV-mediated transgene expression to endogenous. We found that transgenic *CDH23* and *ALMS1* were 102% ± 55% and 7% ± 2% of endogenous, respectively (n = 3 eyes/each group). The apparently different levels of CDH23 and ALMS1 transgene expression result from different levels of their corresponding endogenous transcripts: indeed, endogenous CDH23 is less expressed (0.005% ± 0.002% of glyceraldehyde 3-phosphate dehydrogenase [GAPDH]) than endogenous ALMS1 (0.22% ± 0.02% of GAPDH).

Then, the RNA population emerging following triple AAVs subretinal administration was analyzed by real-time qPCR using primer sets which were previously shown to amplify *in vitro* transcripts with a relative abundance >0.1 of full-length (Figure 3B, “*in vitro*” column). Among them, the following primer resulted in amplification of transcripts with a relative abundance higher than full-length: A + B, A + D, B + C, B + E, C + D, and, only for ALMS1, B + D and E + F (shown in bold in Figure 3B, “mouse retina” column).

**Figure 7:**
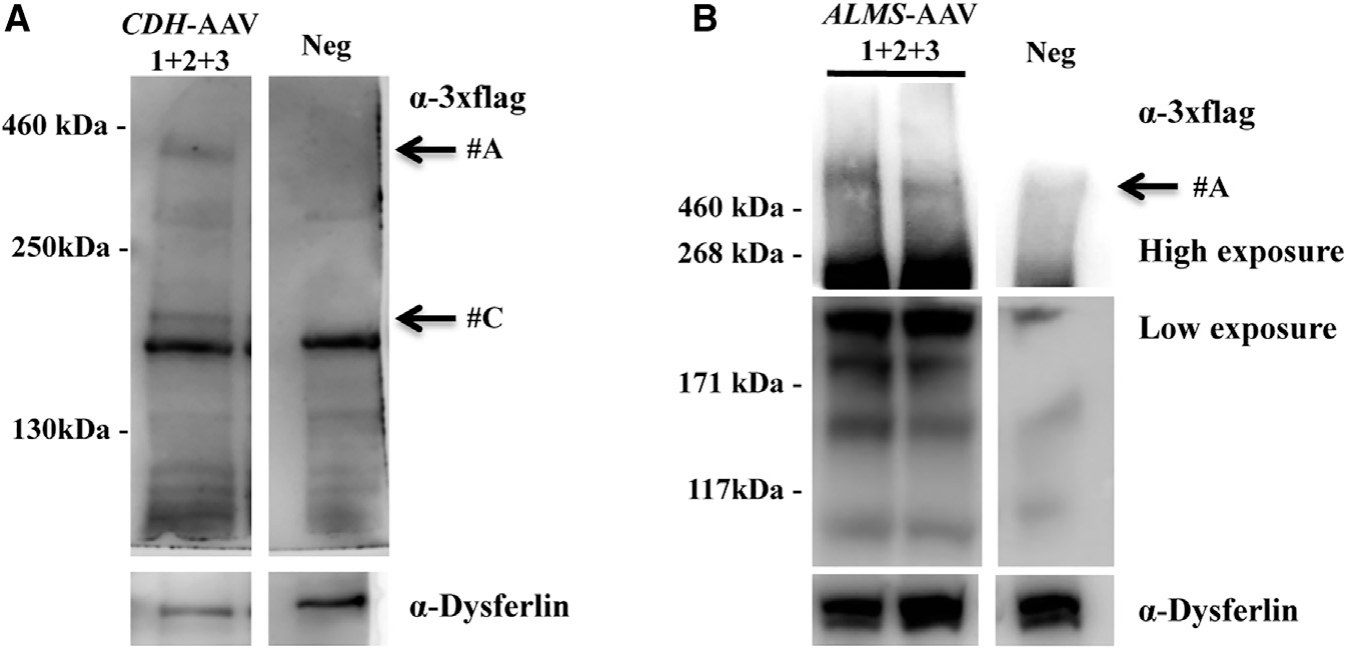
Subretinal Administration of Triple AAV Vectors Results in Full-Length CDH23 and ALMS1 Protein Expression in the Mouse Retina (A) Western blot (WB) analysis of eyecup lysates from C57BL/6J mice 3 months following subretinal injection of triple AAV2/8 encoding for CDH23 under the control of the ubiquitous CMV promoter. The arrows on the right indicate the following products: #A, full-length proteins; #C, probably from AAV 1 + 3. (B) WB analysis of eyecup lysates from C57BL/6J mice 3 months following subretinal injection of triple AAV2/8 encoding for ALMS1 under the control of the ubiquitous short CMV promoter. The arrow on the right indicates full-length ALMS1. α-3xflag, WB with anti-3xflag antibodies; α-Dysferlin, WB with anti-Dysferlin antibodies, used as loading control. Neg, lysates of eyecups following injection with PBS. The molecular weight ladder is depicted on the left; 200 μg of proteins (A) or the whole-eyecup lysates (B) were loaded.

**Figure 8:**
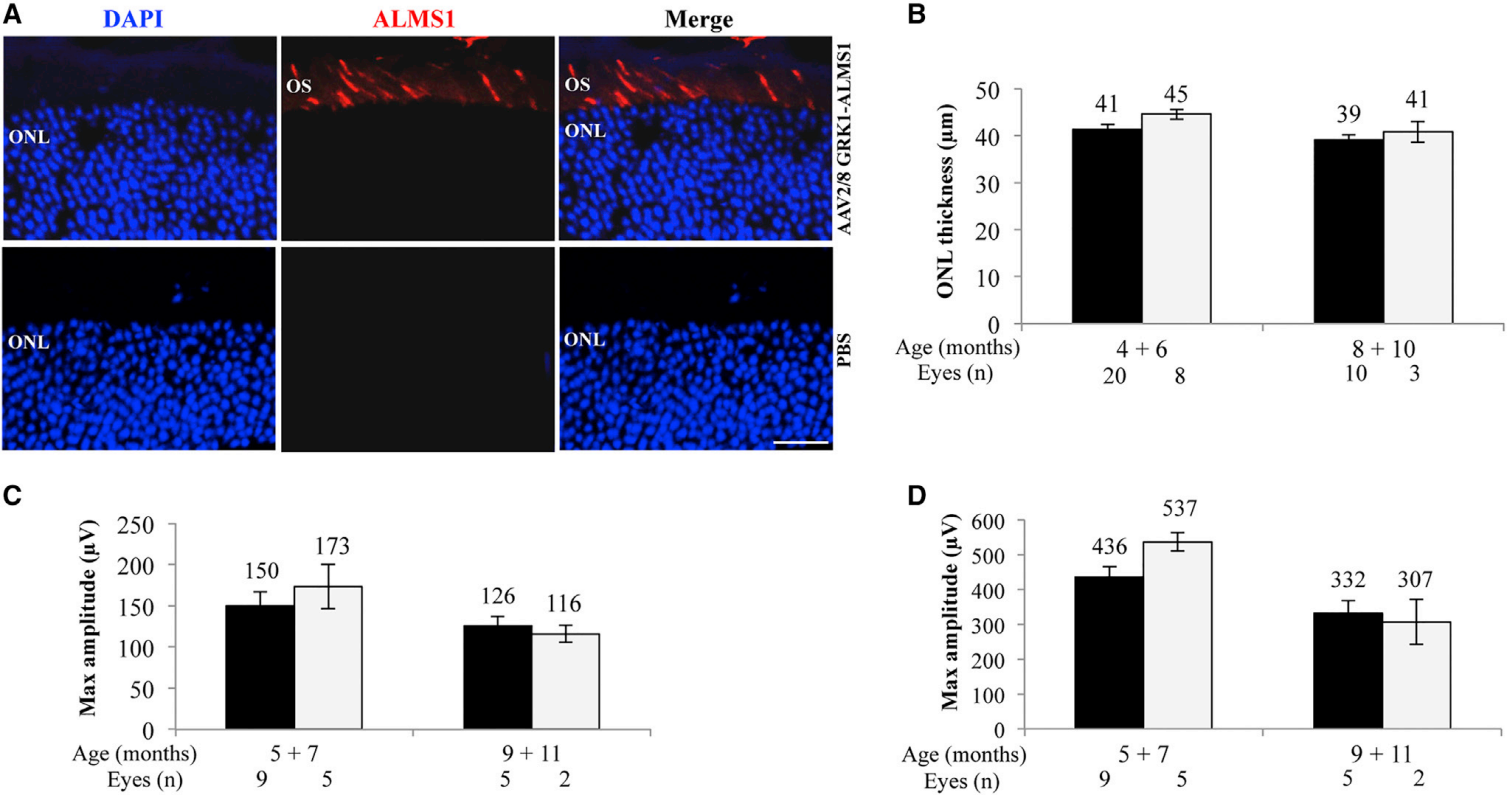
*Alms1*^−*/*−^ Retinal Structure and Function following Subretinal Delivery of Triple AAV Vectors (A) Immunohistochemical (IHC) analysis with anti-3xflag antibodies of retinal cryosections from C57BL/6J mice 2 months following subretinal injections of either triple AAV2/8 encoding for ALMS1 under the control of the PR-specific GRK1 promoter (top panels) or PBS (bottom panels). The pictures are representative of n = 4 injected eyes. The scale bar (20 μm) is depicted in the figure. Merge, overlay of DAPI and ALMS1. ONL, outer nuclear layer; OS, outer segment. (B) Spectral domain optical coherence tomograms (SD-OCT) analysis of *Alms1*^−*/*−^ mice injected subretinally with either triple GRK1-*ALMS*-AAV vectors (white bars) in one eye or PBS in the contralateral eye (black bars). Results are reported as means ± SE. (C and D) Electroretinographic analysis of a-wave (C) and b-wave (D) light responses of *Alms1*^−*/*−^ mice injected subretinally with either triple GRK1-*ALMS*-AAV vectors (white bars) or PBS in the contralateral eye (black bars). Results are reported as means ± SE. Light intensity of 20 cd s/m^2^ (a-wave); background white light of 50 cd s/m^2^ and light intensity of 20 cd s/m^2^ (b-wave).

To understand which of these transcripts were translated to detectable levels, WB analysis of injected retinas was performed using anti-3xflag antibodies: a faint but consistent band corresponding to full-length protein was detected in 11 out of 15 eyes and in 5 out of 8 eyes injected with either *CDH*- (Figure 7A) or *ALMS*-AAVs, respectively (Figure 7B). A shorter product was observed in the CDH23 sample (#C), which is similar to the most intense band present in the *CDH*-AAV 1 + 3 sample *in vitro* (Figure 4A). No truncated products were observed by WB in retinas injected with triple *ALMS*-AAVs. Immunohistochemistry (IHC) analysis of retinal cryosections following subretinal injections with triple *CDH*-AAVs confirmed expression in PRs (data not shown). A similar analysis was performed with the set of triple *ALMS*-AAV vectors used to rescue the retina of ALMS mice (see next paragraph and Figure 8A).

### Subretinal Administration of Triple AAV Vectors in ALMS Mice

Retinal thickness and electrophysiological responses of *Alms1*^−*/*−^ were both reduced compared to littermate control (*Alms1*^−*/+*^ or wild-type) mice starting around 4–6 months (data not shown), similar to what observed in other *Alms1*^−*/*−^ models.^46^, ^47^

To test whether the levels of PR transduction obtained with triple AAV vectors are therapeutically relevant, we have generated triple *ALMS*-AAV vectors that include the GRK1 promoter, which restricts transgene expression to PRs, where ALMS1 is known to localize.^48^, ^49^ Subretinal injection of these vectors (dose of each vector/eye, 2 × 10^9^ GC) in 4-week-old C57BL/6J mice results in ALMS1 proper PR localization 2 months after injection (Figure 8A). We can infer that transgenic ALMS1 staining derives from full-length protein, as ALMS1 potential localization signal is predicted to be at the N terminus of the protein^50^ while the 3xflag tag at the C terminus, and transcripts from *ALMS*-AAV 1 + 3 would be out of frame. Triple GRK1-*ALMS*-AAV vectors were then injected subretinally in 4-week-old *Alms1*^−*/*−^ mice,^51^ which received PBS in the contralateral eye. Four months post-injection, we evaluated by real-time qPCR the expression of transgenic *ALMS1* transcript and found that this was 8.1% ± 6.6% of endogenous. Based on this, we then moved to evaluate their impact on *Alms1*^−*/*−^ retinal morphology and function: in the *Alms1*^−*/*−^ eyes injected with triple vectors we observed a modest, not significant improvement in the outer nuclear layer (ONL) thickness (Figure 8B), and in the electroretinogram a- (Figure 8C) and b-wave (Figure 8D) amplitudes between 5 and 7 months of age (i.e., 4–6 months after gene delivery), which was lost at 9–11 months of age (Figures 8B–8D).

## Discussion

While AAV-mediated gene therapy is effective in animal models and in patients with inherited blinding conditions,^3^, ^4^, ^5^, ^6^, ^7^, ^52^ its application to diseases affecting the retina and requiring transfer of sequences larger than 5 kb is limited by the small cargo capacity of AAV. To overcome this, dual AAV vectors, which exploit AAV genome tendency to concatemerize upon infection,^25^, ^53^, ^54^ have been developed in order to expand AAV DNA transfer capacity to around 9 kb. Here, we demonstrate both *in vitro* and in the mouse and pig retina that similar strategies can be further implemented by including a third vector in the system, thus expanding AAV transfer capacity to approximately 14 kb.

Using the ubiquitous CMV as well as the PR- and RPE-specific IRBP and VMD2 promoters, we showed that full-length gene reconstitution and expression is more efficient in mouse RPE than in PRs. This difference is probably due to a combination of the well-known preferential ability of AAV to transduce RPE than PRs,^9^ as well as of poor triple AAV genome recombination in PRs. Weak promoter activity appears as a less likely explanation, as we observed robust ED expression from the IRBP promoter using a single AAV vector (Figure 5E). This low efficiency in PR transduction with triple AAVs is similar to what we have previously observed with dual AAV vectors,^26^ and is consistent with the low levels of homologous recombination reported in post-mitotic neurons.^45^ It is important to note that the spontaneous fluorescence of the fusion protein ED is weaker than the fluorescence of the single native reporters (Figure S1). Thus, the actual number of PRs transduced by triple AAV vectors may be higher than 3.6% observed in Figure 5A.

The percentage of mouse PR transduction is similar to the percentage of HEK293 cells transduced by triple AAV vectors (2.78% of total cells or 5.96% of transduced cells) despite the 25–50 times lower MOI predicted in the retina than used in *in vitro* infections. Indeed, Ortín-Martínez et al.^55^ estimated that the total number of PRs in a mouse retina is 6.4 × 10^6^. Considering that we transduce, on average, one-third of the retina (which corresponds to about 2 × 10^6^ PRs) we can infer that the MOI in the retina is between 1,000 and 2,000, as opposed to 5 × 10^4^ GC/cell used *in vitro*. The imaging data only apparently differ from what we observe in the WB, where the levels of transgene expression appear weaker *in vivo* than *in vitro* (CDH23, Figure 4A versus Figure 7A; ALMS1, Figure 4C versus Figure 7B). Indeed, the *in vivo* WB analyses are performed on material extracted from whole eyes as opposed to the imaging analysis, which is conducted on the transduced area: thus, in WB, the transgenic protein from the transduced area is diluted within untransduced tissue.

Interestingly, while in mouse retina, ED expression observed with triple IRBP-*ED*-AAV vectors represented only 2% of that obtained with single IRBP-*ED*-AAV, in pig retina this increased to 39%. This is consistent with our previous results that the relative PR-EGFP expression obtained upon subretinal delivery of dual AAV vectors is 6% in mice^26^ and 40% in pigs of that obtained with single AAV, which was explained by the higher PR co-transduction rate of two independent AAVs observed in pigs (74%) than in mice (24%).^56^ This could be due to thicker physical barriers in the pig than in the mouse retina that might limit vector diffusion, thus concentrating the vector in a smaller area and maximizing co-infection of the same cells by multiple vectors. Also, species-specific differences in the efficiency of both AAV2/8 transduction and/or vector intermolecular recombination cannot be excluded. This result is particularly important, considering that the pig retina is an excellent pre-clinical model with a size and architecture more similar to the human than the mouse retina.^10^, ^57^, ^58^, ^59^, ^60^, ^61^

An important concern for clinical use of multiple AAVs is the possible generation of truncated proteins, which might affect both safety and functionality of the treatment. We addressed this important issue by characterizing both transcripts and translated products from triple AAV vectors *in vitro* and in the mouse retina. The *in vitro* real-time qPCR and RNA-seq analyses showed that most of the products expressed at high levels are predicted to be generated by similar AAV genome concatemers between triple *CDH*- and *ALMS*-AAVs (Figure 3). Interestingly, in addition to the products from concatemers containing either simultaneously the promoter and the polyadenilation signal (i.e., AAV 1 + 3 and AAV 1 + 2 + 3) or the promoter alone (AAV 1 with or without AAV 2), we found high levels of expression from AAV 2 in the case of *ALMS1*. It is possible that, in this case, the ITR-mediated promotorial activity^62^ combined with stability of the transcript results in high levels of aberrant transcript. However, the absence of a Kozak consensus sequence with a close-by ATG suggests that this transcript should not be efficiently translated.

Importantly, the translation of the various aberrant transcripts detected appears to be inefficient, as only few truncated products, mostly predicted from AAV 1 + 3 concatemerization, were observed in the WB analysis of cells infected with triple AAVs. One exception appears to be a protein product that is predicted to derive from *CDH*-AAV 3 that was detected, despite its low level of transcription, suggesting a high stability of this product. Interestingly, the various alternative CDH23 isoforms shown by RNA-seq (Figure S2A) may be responsible for the great number of protein products present in *CDH*-AAV 1 + 3 sample. One important phenomenon that we have observed is that most of the truncated products present when only 1 or 2 vectors were used for the infection decreased when the third vector was added (Figures 2A–2C and 4A). This suggests that when evaluating the potential toxicity of triple AAV vectors, this should be done in the context of all vector components and not be extrapolated from combinations of single AAV.

The levels of aberrant proteins observed *in vivo* was even lower than that observed *in vitro* (Figures 5B and 7). This is interesting, as some of the transcripts detected by real-time qPCR in the mouse retinas transduced with triple AAVs were more abundant than in cells (primer sets A + B, A + D, B + C, B + D, C + D, and E + F) and suggests a more stringent protein quality control in live animals than in HEK293 cells. The only exception seems the detection of EGFP-only positive cells in retinal sections of animals injected with *ED*-AAV 1 + 2 + 3 (Figure 5A). This could be the result of products from *ED*-AAV 1 + 2 that are particularly stable and might accumulate over the 2-month time of the *in vivo* but not of the shorter *in vitro* experiments (Figure 2C). The low sensitivity of the WB with anti-EGFP antibodies may explain why the product from *ED*-AAV 1 + 2 is only detected by direct fluorescence analysis of retinal cryosections. In addition, the presence of protein products deriving from alternative open reading frames (ORFs), which would not be detected with the set of antibodies used in this study, cannot be excluded at this point.

In agreement with the low levels of unexpected protein products in the mouse retinas, no evident signs of toxicity were observed by optical coherence tomography analysis (data not shown) in animals injected 2 months before with either *CDH*- or *ALMS*-AAV 1 + 3, the combination of viruses that produced the highest levels of truncated products *in vitro*. Nevertheless, before considering the system for a therapeutic use in humans, more efforts should be focused to evaluate potential toxicity of truncated products, as well as strategies to eliminate them, such as the inclusion of degradation signals strategically placed in the vectors, which we have recently demonstrated to be effective at abolishing selectively truncated proteins from dual AAV vectors.^63^

The possibility to further expand the dual AAV vectors to triple in order to express larger genes has been recently evaluated in mouse muscle^39^, ^40^ for dystrophin transduction, but with poor efficiency. Although a direct comparison between the triple AAV vectors used in that study and our triple AAV vectors is difficult, the levels of full-length transgene expression we achieve in the retina with triple AAV vectors appear to be higher, as we are able to detect full-length protein reconstitution by WB, in addition to fluorescence analysis on histological sections that were used for dystrophin detection in Lostal et al.^39^ and Koo et al.^40^

The expression levels achieved in mice with triple *ED*-AAV compared to single *ED*-AAV vectors are similar to those that we previously observed with dual AAV vectors, both in the mouse and in the pig retina,^26^ suggesting that, in the subretinal space, co-infection by multiple AAV vectors is favored.

If we consider that 3.6% of PRs are transduced by triple AAV vectors following subretinal administration in mice (Figure 5A) and we calculate the transgene expression levels per cell, we find that the 102% of endogenous CDH23 achieved with triple *CDH*-AAVs corresponds to around 3,000% per cell. This is, however, due to the low levels of endogenous CDH23 expression in mice (where it reaches only 0.005% of GAPDH levels) but not in humans^64^ (http://retina.tigem.it). If we, instead, consider ALMS1, whose endogenous levels are higher than CDH23 and reach 0.22% of GAPDH, the levels of recombinant *ALMS1*, which are 7%–8% of endogenous, correspond to approximately 200% per cell. In essence, the levels of *CDH23* and *ALMS1* transgene expression from triple AAV vectors relative to GAPDH are similar, while endogenous ALMS1 is expressed at higher levels than CDH23 in mice.

Mice with ALMS1^46^ but not CDH23^65^ deficiency present with PR degeneration. To understand whether the transduction levels from triple AAV vectors may be therapeutically relevant, we have treated *Alms1*^−*/*−^ mice with subretinal injections of triple GRK1-*ALMS*-AAV vectors, but, besides a transient improvement, we did not observe any significant amelioration of the phenotype. After confirming that transgenic *ALMS1* is properly expressed in PRs at levels around 8% of endogenous, there could be several reasons that justify this lack of therapeutic efficacy: (1) as we have transferred the human *ALMS1* transgene in *Alms1*^−*/*−^ mice, we cannot exclude that interspecies differences might be responsible for low therapeutic efficacy; (2) the non-cell-autonomous PR degeneration described in retinitis pigmentosa^66^ may as well account for the lack of therapeutic effect; and (3) as observed with triple *ED*-AAVs, only 3.6% of PRs are transduced (Figure 5A), and such fraction might not be sufficient to detect a significant rescue. It is possible that higher levels of transduction may improve the efficacy of triple AAV vector. This could be obtained (1) with higher vectors doses, (2) with more efficient AAV serotypes than those used here (i.e., AAV Y444, 500, 730F with mutated tyrosine residues),^67^, ^68^ (3) by enlarging the transduced area with a different injection technique (i.e., double injection) or by intravitreal injection of highly penetrating serotypes such as AAV7m8,^69^ (4) by using a more potent expression cassette than the one used in this work, or (5) by co-injection of AAV transduction enhancers (i.e., proteasome inhibitors).^70^, ^71^, ^72^ Although these levels of rescue appear insufficient in *Alms1*^−*/*−^ mice, it is important to note that, similar to what is observed with dual AAV vectors, the level of pig PR ED transduction by triple versus single AAV2/8 vectors is higher than in mice, which is promising in view of future therapeutic applications, given the closer similarity between human and pig than mouse retina.

Overall, our data demonstrate that triple AAV vectors drive large gene reconstitution both *in vitro* and *in vivo* and that this approach leads to the reconstitution of expression of large genes mutated in IRDs like USHID or ALMS, thus extending AAV transfer capacity up to 14 kb in the retina. This bodes well for the development of triple AAV vectors for other retinal and non-retinal conditions, which require transfer of such large sequences.

## Materials and Methods

### Generation of AAV Vector Plasmids

The plasmids used for AAV vector production derived from the pAAV2.1^73^ plasmids that contain the ITRs of AAV serotype 2.

The *EGFP-DsRed*-AAV (*ED*-AAV) vectors were generated by cloning Discosoma red fluorescent protein (DsRed) CDS (675 bp), from the p*DsRed*-Express2 plasmid (#632535; Clontech, Saint-Germain-en-Laye, France) after the EGFP CDS (717 bp) of pAAV2.1-CMV-*EGFP* plasmid^73^ separated by a 39-bp spacer and followed by a 3xflag sequence and the bovine growth hormone polyadenylation signal (bGHpA).

To generate triple *ED*-AAV vector plasmids, the *ED* CDS was split into three constructs: the first one (*ED*-AAV 1) containing the promoter and the N-terminal *EGFP* CDS^74^ (bp 1–393), the second one (*ED*-AAV 2) containing the C-terminal *EGFP* CDS^74^ (bp 394–717) plus the N-terminal *DsRed* CDS (FJ226077, bp 1–307), and the last one (*ED*-AAV 3) containing the C-terminal *DsRed* CDS (FJ226077, bp 308–675) plus the *3xflag*.

As recombinogenic regions at the 3′ end of *ED*-AAV 1 and 5′ end of *ED*-AAV 2, we used the AK sequence, derived from the phage F1 genome (J02448.1, bp 5,850–5,926), which we previously demonstrated to be effective in the context of dual AAV vectors.^26^ As a second recombinogenic region (at the 3′ end of *ED*-AAV 2 and 5′ end of *ED*-AAV 3), we used a 200-bp sequence (NM_000350.2, bp 3,103–3,302) derived from the human ATP-binding cassette, sub-family A 4 gene (*ABCA4*), which we found to be recombinogenic in dual overlapping AAV vectors (AB200; data not shown).

To generate plasmids for triple *CDH-* and *ALMS-*AAV vectors, the three fragments for each *CHD23* (AF312024.1) and *ALMS1* isoform 1 (NM_015120.4) were obtained by gene synthesis (MWG, now Eurofins Genomics, Ebersberg, Germany). *CDH-* and *ALMS*-AAV 1 contain the N-terminal CDS (*CDH23*, AF312024.1, bp 1–3,369; *ALMS1*, NM_015120.4, bp 112–4,188); *CDH-* and *ALMS*-AAV 2 contain the body of the CDS (*CDH23*, AF312024.1, bp 3,370–6,712; *ALMS1*, NM_015120.4, bp 4,189–8,452); and *CDH-* and *ALMS*-AAV 3 contain the C-terminal CDS (*CDH23*, AF312024.1, bp 6,713–10,065; *ALMS1*, NM_015120.4, bp 8,453–12,618) followed by a 3xflag tag and the bGHpA.

Note that the *CDH23* CDS was split at a natural exon-exon junction, while this was not possible for *ALMS1*, due to the exon length. Moreover, in both systems, fragment 1 and fragment 3 were designed in order to generate out-of-frame transcripts in case of undesired concatemerization.

The AK recombinogenic sequence was placed at the 3′ of both *CDH*- and *ALMS*-AAV 1 and at the 5′ of *CDH*- and *ALMS*-AAV 2. In the *CDH23* expression system, we placed the AP sequence (NM_001632.4, bp 1,802–1,516) derived from the human placental alkaline phosphatase gene^75^ at the 3′ of *CDH*-AAV 2 and at the 5′ of *CDH*-AAV 3, which results in levels of *CDH23* expression higher than with AB200 (data not shown). Due to size constraint, no recombination signals were placed at the 3′ of *ALMS*-AAV 2 nor at the 5′ of the *ALMS*-AAV 3; full-length gene reconstitution, in this case, relies exclusively on ITR-mediated joining followed by splicing.

The splice donor (SD) and splice acceptor (SA) signals contained in triple AAV vector plasmids are as follows: 5′-GTAAGTATCAAGGTTACAAGACAGGTTTAAGGAGACCAATAGAAACTGGGCTTGTCGAGACAGAGAAGACTCTTGCGTTTCT-3′ (SD); 5′-GATAGGCACCTATTGGTCTTACTGACATCCACTTTGCCTTTCTCTCCACAG-3′ (SA).

The ubiquitous CMV promoter is the one contained in pAAV2.1-CMV-*EGFP*;^73^ the ubiquitous shCMV promoter is the one described in Pellissier et al.^76^; the PR-specific human IRBP (X53044.1, bp 2,603–2,837) and the human GRK1 promoter (AY327580.1, bp 1,793–2,087) have been both described to drive high levels of combined rod and cone PR transduction in various species;^14^, ^41^, ^42^ the RPE-specific VMD2 promoter (NG_009033.1, bp 4,870–5,516) corresponds to the previously described EcoRI-XcmI promoter fragment^43^ and was amplified from human genomic DNA. The details of the cloning strategies as well as plasmid sequences are available upon request.

### AAV Vector Production and Characterization

AAV vectors were produced by the TIGEM AAV Vector Core by triple transfection of HEK293 cells followed by two rounds of CsCl_2_ purification.^77^ For each viral preparation, physical titers (GC/mL) were determined by averaging the titer achieved by dot-blot analysis^78^ and by PCR quantification using TaqMan (Applied Biosystems, Carlsbad, CA, USA).^77^ The probes used for dot-blot and PCR analyses were designed to anneal with the promoter for the 5′ vector, the bGHpA region for the 3′ vectors, and within the gene for the body vectors. The length of probes varied between 200 and 700 bp.

### Transfection and AAV Infection of HEK293 Cells

HEK293 cells were maintained in DMEM containing 10% fetal bovine serum (FBS) and 2 mM L-glutamine (Gibco, Thermo Fisher Scientific, Waltham, MA, USA). Depending on the experiment, cells were plated in 6-well plates (1 × 10^6^ cells/well), 24-well plate (2.5 × 10^5^ cells/well), or 8-chamber (1 × 10^5^ cells/well) and transfected 16 hr later with the plasmids encoding for the desired transgene using the calcium phosphate method (1 to 2 μg/1 × 10^6^ cells); medium was replaced 4 hr later.

For AAV infection, plated cells were first transfected with 1.5 μg/1 × 10^6^ cells of pDeltaF6 helper plasmid, which contains the Ad helper genes.^79^ After 4 hr, cells were washed once with serum-free DMEM and incubated with AAV2/2 vectors (MOI 5 × 10^4^ GC/cell of each vector) in a final volume of 700 μL of serum-free DMEM. Two hours later, 1.3 mL of complete DMEM was added to the cells.

To increase transgene expression, 7.5 μM Calpain inhibitor I ([PI] A6185; Sigma-Aldrich, St. Louis, MO, USA), which is known to increase AAV-mediated transduction,^80^ was added either once after infection with triple *ED*- and *CDH*-AAVs or daily after infection with triple *ALMS*-AAVs.

### Animal Models

Mice were housed at the Institute of Genetics and Biophysics animal house (Naples, Italy) and maintained under a 12-hr light/dark cycle. C57BL/6J mice were purchased from Harlan Italy SRL (Udine, Italy).

ALMS (referred as *Alms1*^−*/*−^) mice were imported from The Jackson Laboratory. The mice were maintained by crossing heterozygous females with heterozygous males. The *Alms1*^−*/*−^ mouse harbors the chemically-induced *Alms1* c.1080 + 2T > C splice mutation (exon 6-intron 6 splice junction) predicted to result in a frameshift and truncation of ALMS1.^51^ The genotype of mice was confirmed by PCR analysis on genomic DNA (extracted from the mouse tail tip) followed by DNA sequencing. The primers used for the PCR amplification are as follows:

Fw: 5′-GGTGCACAGAGTGAAAGAATTGC-3′
Rev: 5′-ACTTACCAGTTAAGCCTTGTAGG-3′,

which generate a product of 147 bp that was subsequently sequenced using the Fw primer.

The Large White female pigs (Azienda Agricola Pasotti, Imola, Italy) used in this study were registered as purebred in the LWHerd Book of the Italian National Pig Breeders’ Association and were housed at the Centro di Biotecnologie A.O.R.N. Antonio Cardarelli (Naples, Italy) and maintained under a 12-hr light/dark cycle.

### Subretinal Injection of AAV Vectors in Mice and Pigs

This study was carried out in accordance with the Association for Research in Vision and Ophthalmology Statement for the Use of Animals in Ophthalmic and Vision Research and with the Italian Ministry of Health regulation for animal procedures (Ministry of Health authorization number 147/2015-PR). Surgery was performed under general anesthesia, and all efforts were made to minimize animal suffering. Mice (4 to 5 weeks old) were anesthetized with an intraperitoneal injection of 2 mL/100 g body weight of ketamine/xylazine, then AAV2/8 vectors were delivered subretinally via a trans-scleral trans-choroidal approach, as described by Liang et al.^81^ Eyes were injected with 1 μL of vector solution. The AAV2/8 doses (GC/eye) varied across different mouse experiments, as described in the Results section. PI, at a final concentration of 30 μM, was added to the vector mix in mouse eyes intended for histology, real-time qPCR, and in 3 out of 15 eyes injected with triple *CDH*-AAV vectors and destined for WB analysis.

Subretinal delivery of AAV2/8 vectors to the pig retina was performed as previously described.^10^ Eyes (n = 3) were injected with 100 μL of AAV2/8 vector solution. The AAV2/8 dose was 1 × 10^11^ GC of each vector/eye; thus, co-injection of triple AAV vectors resulted in a total of 3 × 10^11^ GC/eye.

### WB Analysis

Samples (HEK293 cells plated in 6-well plates or eyecups [cups + retinas]) for WB analysis were lysed in radio-immunoprecipitation assay (RIPA) buffer (50 mM Tris-HCl [pH 8.0], 150 mM NaCl, 1% NP40, 0.5% Na-deoxycholate, 1 mM EDTA, 0.1% SDS [pH 8.0]) to extract ED, CDH23, and ALMS1 proteins from HEK293 cells and eyecups. Lysis buffers were supplemented with protease inhibitors (Complete Protease inhibitor cocktail tablets; Roche, Basel, Switzerland) and 1 mM phenylmethylsulfonyl. After lysis, ED, CDH23, and ALMS1 samples were denatured at 99°C for 5 min in 1× Laemmli sample buffer. For ALMS1, 1× Laemmli sample buffer was supplemented with 4 M urea. Lysates were separated by 12% (ED samples), 6% (CDH23 samples), or gradient (#4561086; Bio-Rad, Hercules, CA, USA) 4%–15% (ALMS1 samples) SDS-PAGE.

The antibodies and dilutions used for immunoblotting are as follows: anti-EGFP (1:1,000, ab1218; Abcam, Cambridge, UK); anti-3xflag (1:1,000, A8592; Sigma-Aldrich, St. Louis, MO, USA); anti-CDH23 (1:500, sc-166005, Santa Cruz Biotechnology, Dallas, TX, USA); anti-ALMS1 (1:1,000, polyclonal; ab4306, Abcam, Cambridge, UK); anti-β Tubulin (1:3,000, T5201; Sigma Aldrich, St. Louis, MO, USA); anti-Filamin A (1:1,000, #4762; Cell Signaling Technology, Danvers, MA, USA); anti-Dysferlin (1:500, Dysferlin, clone Ham1/7B6, MONX10795; Tebu-bio, Le Perray-en-Yveline, France); anti-β-Actin (1:1,000, NB600-501; Novus Biological, Littleton, CO, USA).

Notably, the epitope to which the monoclonal anti-EGFP antibodies were raised has not been disclosed by the company; however, we deduced that it maps to the C terminus of EGFP based on the pattern observed in the WB analysis in Figure 2B.

The quantification of ED bands detected by WB was performed using ImageJ software (http://rsbweb.nih.gov/ij/); to compare data across different experiments, in each WB, the average intensity of the bands deriving from single AAV-transduced eye was set as 1, and the intensity of triple AAV transduced eye was divided by that of single AAV-transduced eye and calculated accordingly.

### Histology and Light and Fluorescence Microscopy

To evaluate ED expression *in vitro*, HEK293 cells, plated in 8-chambers at a density of 1 × 10^5^, were infected as previously described. Seventy-two hours post-infection, cells were washed once with PBS, fixed for 7 min with 4% paraformaldehyde (PFA) in PBS, washed three times with PBS, and mounted with Vectashield with DAPI (Vector Lab, Peterborough, UK). Cells were analyzed under the Axio Observer Z1 (Carl Zeiss, Oberkochen, Germany) equipped with ZEN software (Carl Zeiss) and using appropriate excitation and detection settings for EGFP, DsRed, and DAPI.

To evaluate transgene expression in histological sections, C57BL/6J mice were injected subretinally with triple AAV vectors (supplemented with PI at a final concentration of 30 μM). Two months later, mice were sacrificed and eyes were fixed in 4% paraformaldehyde overnight and infiltrated with 30% sucrose overnight; the cornea and the lens were then dissected, and the eyecups were embedded in optimal cutting temperature compound (O.C.T. matrix; Kaltek, Padua, Italy). Ten-micrometer-thick serial retinal cryosections were cut along the horizontal meridian, progressively distributed on slides, and mounted with Vectashield with DAPI (Vector Lab, Peterborough, UK). Then, the cryosections were analyzed under the confocal LSM-700 microscope (Carl Zeiss, Oberkochen, Germany) using appropriate excitation and detection settings.

For assessment of PR transduction in mouse retinal cryosections following triple *ED*-AAV administration, a single section/eye within the transduced area was selected; the whole slice was acquired at 40× magnification and then analyzed using ImageJ software (http://rsbweb.nih.gov/ij/). A minimum of 600 PRs, identified by DAPI staining, were counted for each eye. PRs with signal compatible with EGFP and DsRed co-expression were unequivocally identified based on their identical shape on picture micrographs of the same field.

In the case of fluorescent IHC staining of ALMS1, sections were washed in PBS for 10 min and were permeabilized and blocked with 0.3% Triton X-100, 5% next-generation sequencing (NGS), 3% BSA in PBS for 3 hr, then the sections were pre-treated with Avidin/Biotin (SP-2001, Vector Lab, Peterborough, UK) according to manufacturer’s instructions. Sections were incubated overnight with anti-3xflag antibodies (1:200 F1804 Sigma-Aldrich, St. Louis, MO, USA). Endogenous peroxidase were blocked with 0.3% hydrogen peroxide (H1009, Sigma-Aldrich, St. Louis, MO, USA). Signal was developed using the Vectastain ABC kit (PK-6200 Vector Laboratories, CA, USA) followed by the SuperBoost Tyramide signal amplification (B40942, Thermo Fisher Scientific, Waltham, MA, USA) according to the manufacturer’s instructions. Stained sections were mounted with Vectashield with DAPI (Vector Laboratories, CA, USA). Cryosections were analyzed under the confocal LSM-700 microscope (Carl Zeiss, Oberkochen, Germany) and acquired at 63× magnification.

### Cytofluorimetric Analysis

HEK293 cells, plated in 6- or 24-well plates, were washed once with PBS, detached with trypsin 0.05% EDTA (Thermo Fisher Scientific, Waltham, MA USA), washed twice with PBS, and resuspended in PBS 5% FBS, 2.5 mM EDTA. Cells were analyzed on a BD FACS ARIA III (BD Biosciences, San Jose, CA, USA) equipped with BD FACSDiva software (BD Biosciences) using appropriate excitation and detection settings for EGFP and DsRed. Thresholds for fluorescence detection were set on not infected cells, and a minimum of 10,000 cells/sample were analyzed.

### RNA Extraction, cDNA Production, and Reverse Transcription Analysis

Total RNA was extracted using the RT-PCR RNeasy MiniKit (QIAGEN, Milan, Italy) from either HEK293 cells plated in 6- or 24-well plates and infected or not with triple AAV vectors or mouse eyecups or retina. RNA (500 ng from cells or eyecups and 360 ng from retina) was submitted to DNase I digestion (RNase Free DNase set; QIAGEN) and 20 μL cDNA was generated using the SuperScript III Reverse Transcriptase kit (Thermo Fisher Scientific, Waltham, MA, USA) using oligo dT primers. For each sample, the same amount of RNA did not receive the retrotranscriptase enzyme and was used as a control for genomic DNA contamination. Primers for real-time qPCR were designed using the bioinformatic program “Primer Blast” (https://www.ncbi.nlm.nih.gov/tools/primer-blast/) and purchased from Eurofins Genomics (Eurofins Genomics, Ebersberg, Germany; Table S1). Serial dilutions of the cDNAs obtained from cells infected with either triple *CDH*- or *ALMS*-AAV 1 + 2 + 3 were used to measure the primer efficiency, which was between 1.75 and 2.25 for all primer sets shown in Figure 3B. SybrGreen real-time qPCR kit was purchased from Roche (Roche, Monza, Italy) and used following the manufacturer’s protocol on the LyghtCycler 96 system (Roche). Five microliters of diluted cDNA (1:25 to 1:50 for *in vitro* experiments and 1:5 for the *in vivo* experiments) and 10 pmol of primers were used for PCR in a total volume of 20 μL. Thermal cycling for all genes initiated with an initial denaturation step at 95°C for 5 min, followed by 45 cycles with denaturation at 95°C for 10 s, annealing at 60°C for 20 s, and extension at 72°C for 20 s. Expression data were then normalized versus the corresponding housekeeping genes (h-β-ACTIN for HEK293 cells and mouse GAPDH (mGAPDH) for eyecups and retinas).

### RNA-Seq Libraries Preparation and Sequencing

A total of 47.5 ng of RNA was used as input for the synthesis of cDNA with the SMART-Seq v4 Ultra Low Input RNA Kit for Sequencing (Takara Bio USA, Mountain View, CA, USA). Manufacturer suggested protocol was followed, with minor modifications. The second strand synthesis of cDNA was performed by replacing the SMART-Seq v4 Oligo with custom primers specific for the transgenes of interest (CDH23 oligo, 5′-AAGCAGTGGTATCAACGCAGAGTacttgttgatctccgaagataccc-3′; ALMS1 oligo, 5′-AAGCAGTGGTATCAACGCAGAGTtagtggtggaggaagtagaggag-3′ [bp in capital letters belong to the RNA-seq adaptor]).

Seventy-five pg of cDNA generated with SMART-Seq v4 Kit were used for library preparation using the NEXTERA XT DNA Library Preparation kit (Illumina, San Diego, CA, USA), following the suggested protocol.

Samples were sequenced using NextSeq 500/550 Mid Output v2 kit in a 150 + 150 paired-end run. The data were deposited in GEO: GSE107173.

### RNA-Seq Analysis

Sequence reads were trimmed using Trim Galore! software (https://www.bioinformatics.babraham.ac.uk/projects/trim_galore/) to remove adaptor sequences and low-quality end bases and then analyzed using the “new tuxedo” software suite.^82^ The idea was to generate a custom reference with a “genomic” sequence of the three vector inserts where to align the reads. The expected full-length transcript was used as known reference, but the aligner software was allowed to introduce new splicing junctions and generate also alternative transcripts if supported by reads (Figure S2).

First, the custom reference sequence was built to perform the viral vector library sequences alignment. For each transgene, the sequence of the three vector inserts were pasted sequentially side by side, separated by a stretch of N bases as spacers to simulate a “genomic” sequence and then added to the human genome (build hg19) as separate contigs. The ITR ends were not included to reduce the possibility of aberrant splicing junctions due to mis-aligned repeated reads. The relative coordinates of the actual CDSs of each transgene were used as reference of a single transcript composed of three exons. The first exon start and the last exon end bases were defined to coincide respectively with the beginning of the specific PCR primer and the expected transcription stop signal, in order to reproduce the expected drop in coverage of the aligned reads due to library construction, to minimize the generation of alternative predicted transcripts only differing from the reference by the start or end coordinates.

Then the analysis was performed following the protocol described in Pertea et al.^82^ Reference-guided alignment was performed with HISAT2^83^ followed by transcript assembly with Stringtie.^84^ The assembly generated for each sample can be slightly different due to the actual coverage of exons and reads spanning exon-exon junctions, so the individual assemblies of each sample were merged into a common consensus with the Stringtie merge function to make them comparable. The expression levels of each transcript of the assembled consensus were then estimated with a final run of Stringtie used to count reads supporting each transcript without allowing the annotation of any further isoform. The R/Biocondictor package Ballgown^85^ was then used to visualize results and extract the raw counts and normalization was performed with DESeq.^86^

To estimate the relative abundance of each annotated alternative isoform, their normalized counts were divided by the corresponding full-length one and the ratios were averaged among three replicate samples.

### Spectral Domain Optical Coherence Tomography

Spectral domain optical coherence tomography (SD-OCT) images were obtained using the Bioptigen Spectral Domain Ophthalmic Imaging System (SDOIS; Envisu R2200, Bioptigen, Morrisville, NC, USA). *ALMS* mice were anesthetized and pupils were dilated by applying 1–2 drops of topical 0.5% tropicamide (Visufarma, Rome, Italy). To prevent corneal desiccation during the procedure, topical lubricant eye drops (Recugel; Bausch & Lomb, Rochester, NY, USA) were applied bilaterally with a small brush. Mice were positioned into the animal imaging mount and rodent alignment stage (AIM-RAS; Bioptigen, Morrisville, NC, USA); the laser source was placed in front of the mouse, and images were acquired by the InVivoVue Clinic software (Bioptigen, Morrisville, NC, USA). Three images, one central, one superior, and one inferior to the optic nerve, were taken from each eye. ONL thickness was manually measured three times from each OCT scan image and averaged.

### Electrophysiological Recordings

For electroretinographic analyses, *ALMS* mice were dark-adapted for 3 hr. Mice were anesthetized and positioned in a stereotaxic apparatus, under dim red light. Their pupils were dilated with a drop of 0.5% tropicamide (Visufarma, Rome, Italy), and body temperature was maintained at 37.5°C. Light flashes were generated by a Ganzfeld stimulator (CSO, Costruzione Strumenti Oftalmici, Florence, Italy). The electrophysiological signals were recorded through gold-plate electrodes inserted under the lower eyelids in contact with the cornea. The electrodes in each eye were referred to a needle electrode inserted subcutaneously at the level of the corresponding frontal region. The different electrodes were connected to a two-channel amplifier. After completion of responses obtained in dark-adapted conditions (scotopic), the recording session continued with the purpose of dissecting the cone pathway mediating the light response (photopic). To minimize the noise, different responses evoked by light were averaged for each luminance step. The maximal scotopic response of rods and cones was measured in dark conditions (scotopic) with two flashes of 0.7 Hz and a light intensity of 20 cd s/m^2^, photopic cone responses were isolated in light conditions with a continuous background white light of 50 cd s/m^2^, with 10 flashes of 0.7 Hz and a light intensity of 20 cd s/m^2^.

## Author Contributions

The study was conceived, designed, and written by A.A., A. Maddalena, and P. Tornabene. All data were generated by A. Maddalena, P. Tornabene, and R.M. except the *Alms1* mouse phenotyping, which was performed by P. Tiberi, and the RNAseq experiments, which were performed by D.C., A. Manfredi, and M.M. S.R. and F.S. performed subretinal injections in pigs. J.K.N. provided the *Alms1*^−*/*−^ mice.

## Conflicts of Interest

The authors declare no conflict of interest.

## Supplemental Information

Document S1. Figures S1–S4 and Table S1

Document S2. Article plus Supplemental Information
