# Supplementary material for: Triple Vectors Expand AAV Transfer Capacity in the Retina
Source: Mol Ther. 2017 Dec 5;26(2):524–41. doi: 10.1016/j.ymthe.2017.11.019 (PMC5835116; doi:10.1016/j.ymthe.2017.11.019)
Supplement: Document S1. Figures S1–S4 and Table S1 [file mmc1.pdf]

YMTHE, Volume *26*

Supplemental Information

Triple Vectors Expand AAV Transfer Capacity in the Retina

Andrea Maddalena, Patrizia Tornabene, Paola Tiberi, Renato Minopoli, Anna Manfredi, Margherita Mutarelli, Settimio Rossi, Francesca Simonelli, Jurgen K. Naggert, Davide Cacchiarelli, and Alberto Auricchio

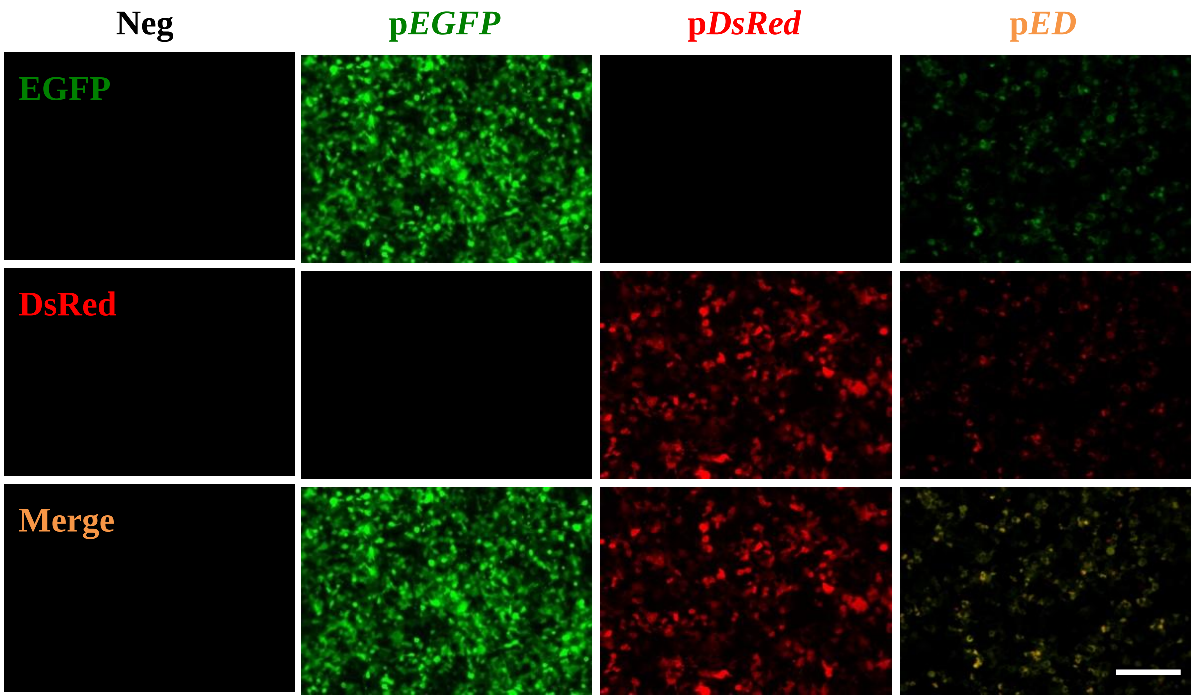

Figure S1. The EGFP-DsRed-3xflag (ED) protein is less fluorescent than the corresponding native EGFP and DsRed reporters.
Fluorescence analysis of HEK293 cells transfected with plasmids encoding either native EGFP (p*EGFP*), native DsRed (p*DsRed*) or the EGFP-DsRed-3xflag reporter protein construct (p*ED*). EGFP, native EGFP fluorescence; DsRed, native DsRed fluorescence; merge: overlay of EGFP and DsRed. The scale bar (200 µm) is depicted in the figure.

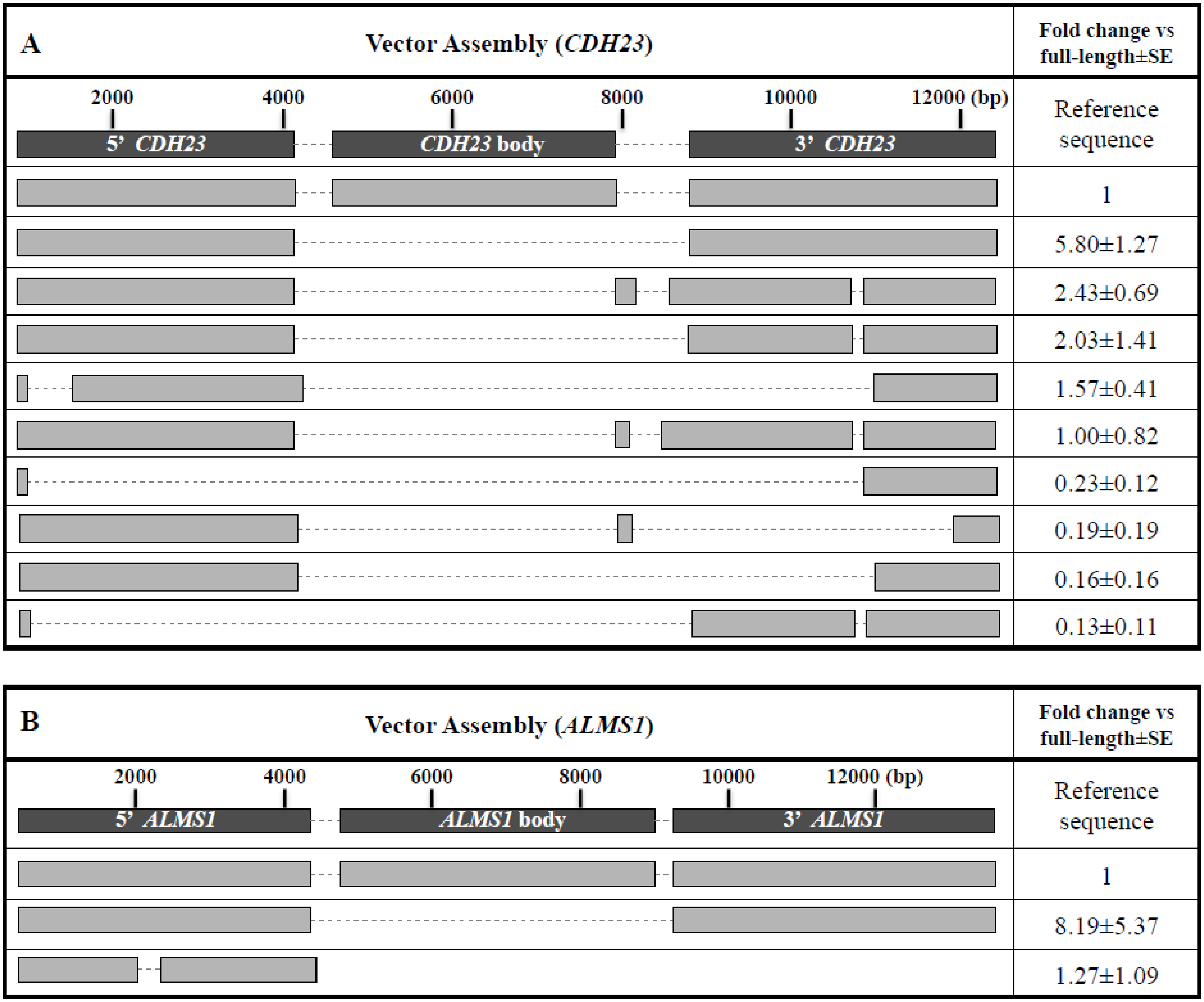

Figure S2. Alternative transgene isoforms annotated by RNA-Seq analysis.
For each transgene, the alternative isoforms are plotted below the full-length transcript. The relative abundance is quantified by mean and standard error of the normalized count ratio vs the full-length among 3 replicates.

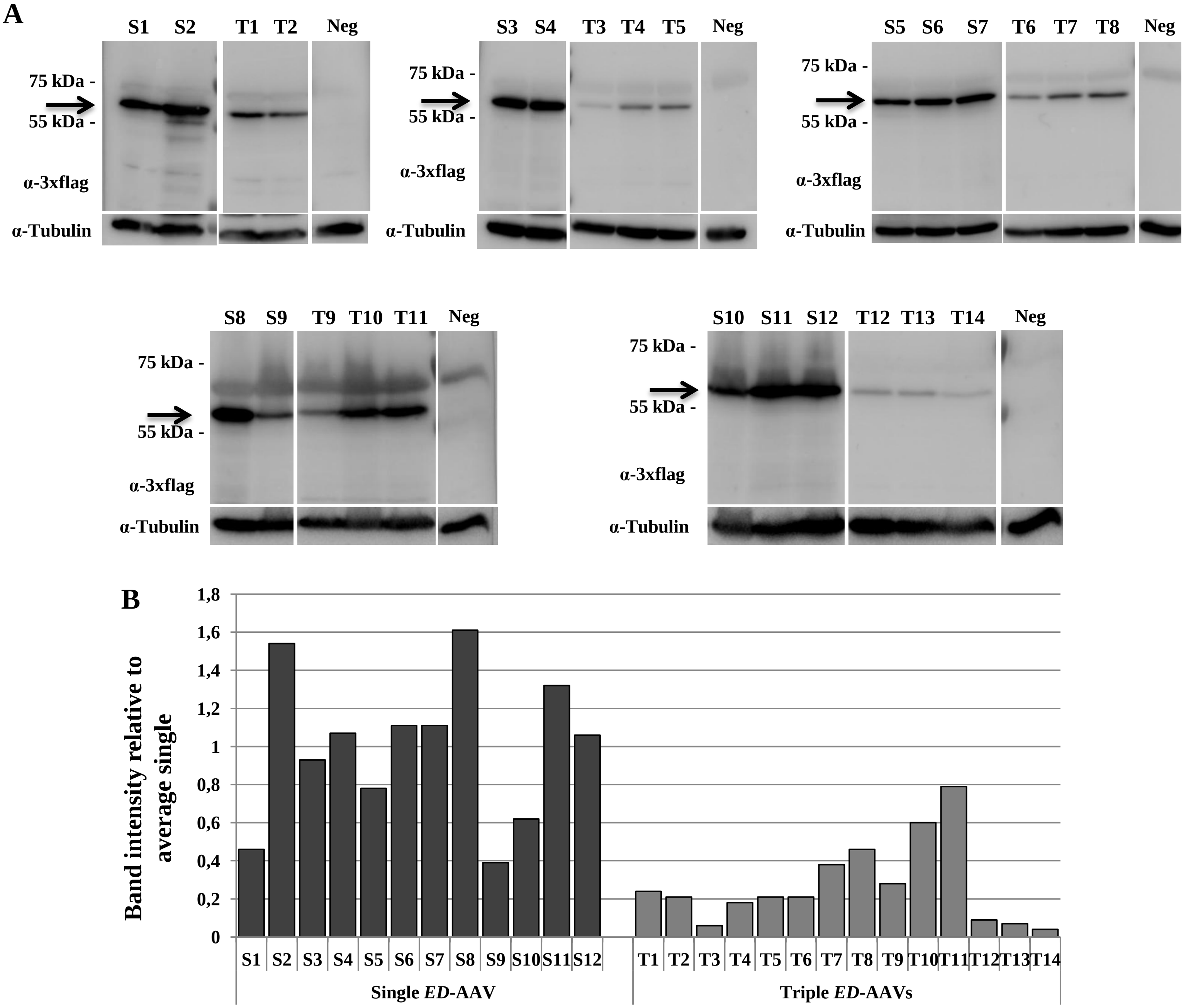

Figure S3. Comparison between single and triple CMV-*ED*-AAV2/8 transduction in mouse retina.

(A) WB analysis of eyecup lysates from C57BL/6J 2 months following subretinal injection of single and triple AAV2/8 vectors encoding for ED under the control of the ubiquitous CMV promoter. α-3xflag, WB with anti-3xflag antibodies; α-β-Tubulin, WB with anti-β-Tubulin antibodies, used as loading control. Neg, lysates from eyecups following injection with PBS. The molecular weight ladder is depicted on the left, 100-150 µg of proteins were loaded. The arrow on the left indicates the full-length ED. S1-12: lysates from eyecups injected subretinally with single CMV-*ED*-AAV: T1-14: lysates from eyecups injected subretinally with triple CMV-*ED*-AAVs. Neg, lysates of eyecups following injection with PBS.

(B) Densitometric values of ED bands in (A). To compare data across different experiments, in each WB the average intensity of the bands deriving from single AAV transduced eye was set as 1 and the intensity of triple AAVs transduced eye was divided by that of single AAV transduced eye and calculated accordingly. S1-12: lysates from eyecups injected subretinally with single CMV-*ED*-AAV. T1-14: lysates from eyecups injected subretinally with triple CMV-*ED*-AAVs.

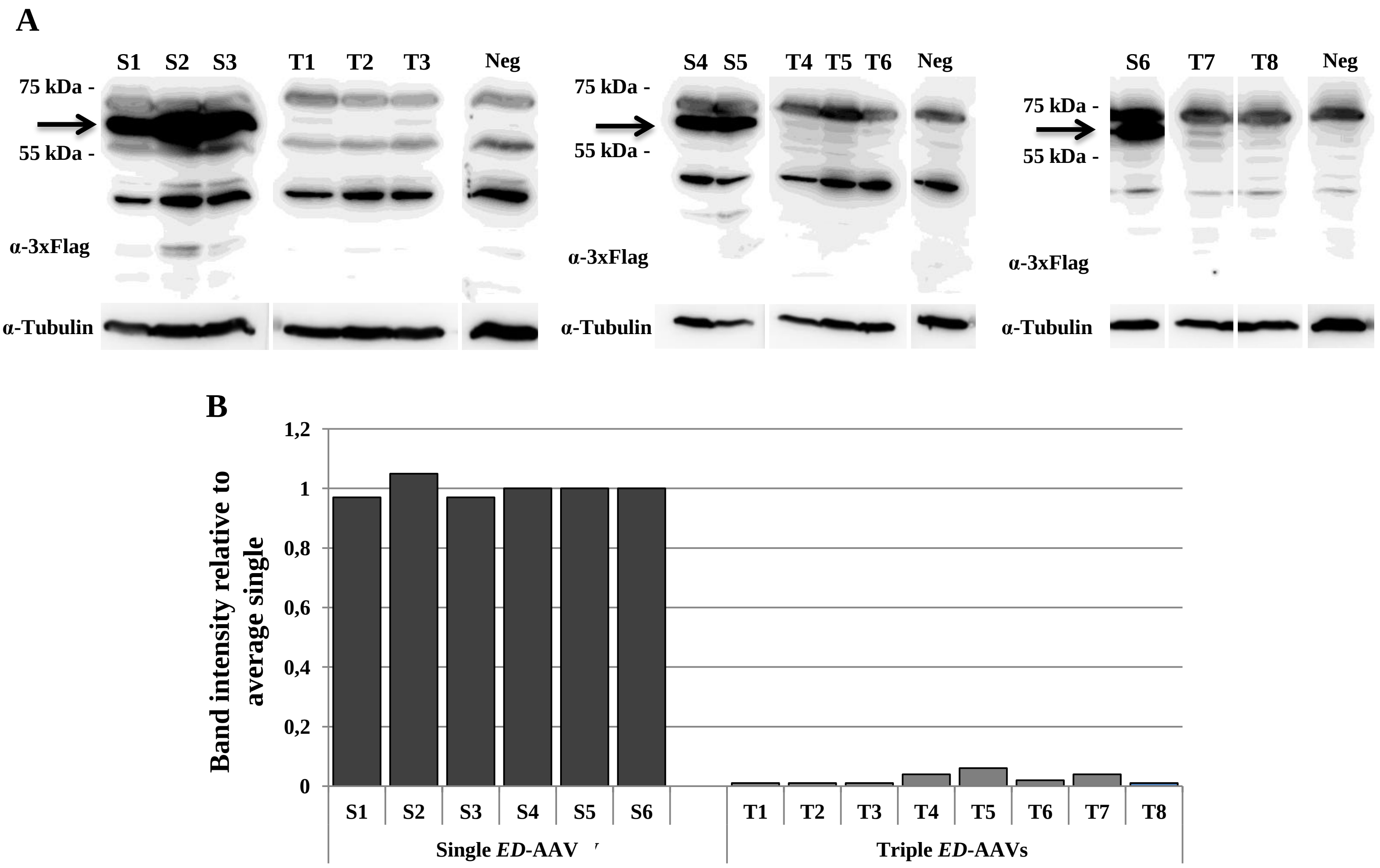

Figure S4. Comparison between single and triple IRBP-*ED*-AAV2/8 transduction in mouse retina.
(A) WB analysis of eyecup lysates from C57BL/6J 2 months following subretinal injection of single and triple AAV2/8 vectors encoding for ED under the control of the PR specific IRBP promoter. α-3xflag, WB with anti-3xflag antibodies; α-β-Tubulin, WB with anti-β-Tubulin antibodies, used as loading control. Neg, lysates from eyecups following injection with PBS. The molecular weight ladder is depicted on the left, 200 µg of proteins were loaded. The arrow on the left indicates the full-length ED. S1-6, lysates from eyecups injected subretinally with single IRBP-*ED*-AAV. T1-8, lysates from eyecups injected subretinally with triple IRBP-*ED*-AAVs. Neg, lysates of eyecups following injection with PBS.
(B) Densitometric values of ED bands in (A). To compare data across different experiments, in each WB the average intensity of the bands deriving from single AAV transduced eye was set as 1 and the intensity of triple AAVs transduced eye was divided by that of single AAV transduced eye and calculated accordingly. S1-6: lysates from eyecups injected subretinally with single IRBP-*ED*-AAV. T1-8: lysates from eyecups injected subretinally with triple IRBP-*ED*-AAVs.

**Primer name**	**Sequence**
CDH A	ctgccaactggggtatcttc
CDH B	ctgttggtaagcggcatact
CDH C	tcagcacgtgactggaattt
CDH D	acatcgtcgggattgtagcga
CDH E	gtaagtggccaccagttccct
CDH F	agaccatgacggcgactata
mCDH wt Forw	catcgtggccaaggatgacac
mCDH wt rev	gaatgctctgggaggtcgct
ALMS A	ctcctctacttcctccaccac
ALMS B	cttctacagccacaccgaga
ALMS C	tgcctcttcagtcagatggg
ALMS D	gccagcgtaggtgtgttcaa
ALMS E	cttctggctttgccttcgtc
ALMS F	aggttccttgggacgactac
mALMS wt forw	taggacgtgaccgtggatact
mALMS wt rev	gccttgtggacttcgtaagc
hβ-ACTIN forw	gcgagaagatgacccagatc
hβ-ACTIN rev	ggatagcacagcctggatag
mGAPDH forw	agcaaggacactgagcaagag
mGAPDH rev	gcagcgaactttattgatggt

Table S1.
List of primers used for Real-Time quantitative PCR. Fw: forward primer; Rev: reverse primer; wt: wild type.
